# Characterising people–species–place relationships

**DOI:** 10.1007/s13280-026-02411-0

**Published:** 2026-05-23

**Authors:** Edith Shum, Vanessa M. Adams, Georgina G. Gurney

**Affiliations:** 1 School of Geography, Planning, and Spatial Sciences, University of Tasmania, Churchill Avenue, Sandy Bay, Hobart, TAS 7005 Australia; 2 Centre for Marine Socioecology, University of Tasmania, Hobart, Australia; 3 College of Arts, Society and Education, James Cook University, 1 James Cook Dr, Douglas, Townsville, QLD 4814 Australia

**Keywords:** Connection to nature, Cultural keystone species, Human–nature relationships, Place attachment, Placemakers, Species attachment

## Abstract

Place attachments are multifaceted, reflecting personal meaning, and lived experiences. People also develop strong connections with species that inhabit beloved places. Yet, where places and species are experienced together, little is known about how these attachments intersect or vary across species. Here, we explored attachments to place and species, focusing on fairy penguins (*Eudyptula minor*) and muttonbirds (*Ardenna tenuirostris*) in their coastal habitat at an iconic Tasmanian landmark (Bruny Island). Using a mixed-methods approach, we examined these relationships through a modified Inclusion of Self Scale (*n* = 93) and interviews (*n* = 28). Species–place relationships, defined as the perceived belonging of a species to a place, emerged as the strongest predictor of place attachment. Qualitative insights revealed species–specific pathways, with connections strengthened when species contributed in both ecological and cultural ways. Recognising these diverse pathways offers a more nuanced understanding of how people–place relationships are formed and sustained.

**Graphical abstract:**

**Figure:**
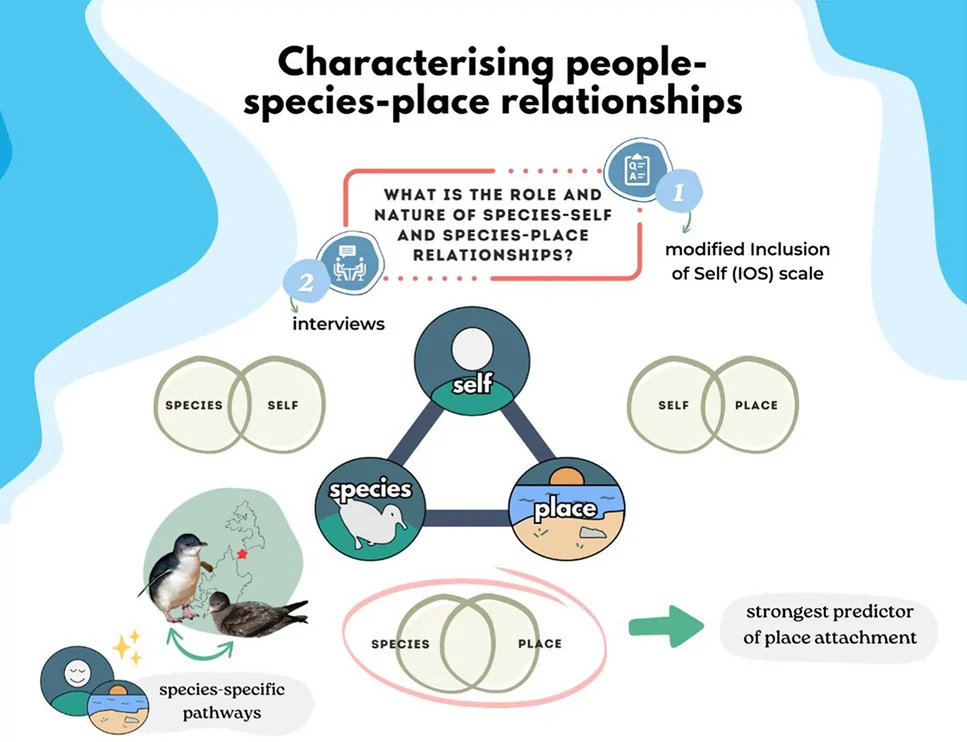

**Supplementary Information:**

The online version contains supplementary material available at https://doi.org/10.1007/s13280-026-02411-0.

## Introduction

Humans form deep, multifaceted bonds not only with other people but also with the natural world, including particular places and favourite species. In the relationships that people hold with places, a large body of literature identifies emotional, functional, and symbolic bonds to place can influence identity and belonging (Williams and Vaske 2003; Scannell and Gifford 2010; Lewicka 2011). Similarly, a parallel body of research has explored people’s attachment to species, finding that perceived similarity, ecological knowledge, and cultural significance all shape the strength and meaning of these bonds (Garibaldi and Turner 2004; Lorimer 2007).

When considering the specific attributes that determine a natural environment, it is difficult to overlook the complex assemblage of species interacting, functioning, and shaping ecological processes together (Loreau 2010; Masterson et al. 2017). In these ecosystems, the structural and functional roles that species play such as those that are engineers, indicators, or culturally significant, emerge as the distinctive character of a landscape (Gobster et al. 2007; von Hellermann 2016; Brazier et al. 2021). Schebella et al. (2017) noted that when people described what they loved most about natural environments, micro-variables, or site-specific small-scale attributes, such as birds and plants, emerged as the two most frequently mentioned elements. This underscores the value that individuals place on the presence of living species in loved environments. In this sense, people’s connection to place are inherently entangled with the species that inhabit it. As people come to understand and notice how species and place are interconnected, these relationships become more strongly reinforced and intertwined in a place-specific way (Hamlin and Richardson 2022). This reflects a reciprocal process whereby attachment to place may foster the valuing of species within that place, while the presence of species may, in turn, shape or reinforce place attachment.

While these reciprocal relationships between people, place, and species, have individually been studied through diverse disciplinary lenses, few studies have examined the role of species in place attachment, or how attachments to species and place interact to co-construct meaning (Shum et al. 2025). Here we emphasise that places are more than reflections of their physical form; they are cultural and symbolic landscapes where species are emerging as key anchors in shaping people’s emotional relationships with place. Therefore, it stands to reason that people, place, and species hold deeply intertwined meanings through a triadic relationship. This underscores the growing need to understand the experiential qualities that make species fundamental to the place experience. To address this gap, this study looks to characterise the relationships between species and place attachment, not only in terms of their strength, but also through the diverse pathways and experiences that underpin them.

### People and place

Our connection to places is often described through the concept of place attachment—the bonds people form with particular settings (Agnew 1987; Malpas 2018). This goes beyond a site’s physical attributes, and also considers the emotions, meanings, and values that people associate with it (Williams and Vaske 2003). These emotional and symbolic bonds often emerge through direct experiences in a place and their association with affective, pleasurable memories, which makes the place an “anchor of [people’s] identity” (Escalera-Reyes 2020). In this sense, connection to place is fluid and shaped uniquely for each person by the particular setting (Scannell and Gifford 2010).

In nature-based contexts, our connection to place is emphasised by the symbolic and relational qualities of nature. For example, natural environments often hold cultural significance, with attachment derived through shared heritage, stories, and traditional practices that foster communal identities and a sense of belonging (Ainsworth et al. 2019). In environmental psychology, these connections are often described as relational, meaning that people build relationships with places through “values associated with living a good life” (Klain et al. 2017). Relational values resonates with nature bonding because connections tend to emerge from specific interactions with the nonhuman environment, including acts of care, responsibility and community practices (Klain et al. 2017; Haywood et al. 2021). As Beery and Wolf-Watz (2014) describes, “….environmental connectedness perspective may suffer from dualistic thinking and a reliance on simple causality”. This explains that nature should not be treated as a standalone entity from a place context, nor should exposure to nature be assumed to drive connectedness. Instead, framing place through relational values moves beyond the material or objective view of nature and instead emphasises the subjective dimensions of place-specific processes. This includes both physical and socio-cultural contexts through which people engage with the natural world. Recognising these relational qualities clarifies how attachment to place is formed and why a relational values framing matter.

### People and species

Just as places carry relational significance, so too can our connection to species demonstrate emotional, cultural, and symbolic depth. This is argued to have stemmed from our shared evolutionary history, as well as from people’s innate desire to understand and relate to the natural world, a concept referred to as biophilia (Fromm 1992). For some, attachment to species arises through perceived anthropomorphic traits—attributing human-like qualities to animals, that make it easier for people to relate to them on a personal level (Vining 2003; Lorimer 2007). For others, our connection to species is shaped by cultural heritage or practices that frame species as part of the community’s identity (Cristancho and Vining 2004). When species are represented in ways that remind us of ourselves, whether that is perceiving a dolphin to display a sense of fun during an interaction (Yerbury and Boyd 2018), or through cultural narratives where they are relied upon as food, medicine or in ceremonial roles (Garibaldi and Turner 2004), people are more likely to form empathetic and meaningful connections with them.

In practice, these connections to species often lead to classifications like “flagship”, “keystone”, or “iconic species where they become a powerful symbol used as a strategic conservation tool for public attention (Jepson and Barua 2015). These classifications not only elevate species as a focal point for conservation efforts but also stimulate curiosity and a desire to know more about them (Lindemann‐Matthies 2005). Similar to the “noticing nature cycle” (Garfinkel et al. 2024), recognising wildlife is argued to be a key entry point for forming deeper connections with the natural world, which can in turn improve wellbeing and pro-conservation attitudes (Soga et al. 2025). In this way, species may evoke personal memories of encounters, serve as cultural symbols embedded in stories or traditions, or represent ecological knowledge that informs how people understand their environment ( Krasny et al. 2014; Härtel et al. 2023). Therefore, our connection to species, like place attachments, extend beyond appreciation of physical presence but also include pathways to which individuals can reflect the values, meanings, and identities that people build in the natural environment.

### Linking people, place, and species

If our connection to place is an integral part to people’s identity, and our connection to species is an innate desire to relate to the natural world, an important question is raised: how do these two dimensions interact? Despite growing interest in both species and place attachment, little is known about how they are interconnected and the ways these links might be maximised to frame conservation and stewardship behaviours. Most existing measures treat them as separate domains, often assessed through lengthy multi-item scales or open-ended interviews (Shum et al. 2025). By treating species and place attachment as separate entities, it overlooks how species and places are experienced together within the natural environment. For example, the presence of particular species can shape the meaning of a place, while attachment to place can also deepen care or concern for its species. Lavy and Zavar (2023) demonstrate this by showing how the loss of culturally significant trees following a natural disaster elicited emotional distress among those strongly attached to the area, which in turn, prompted collective action to rebuild and restore the urban forest. Indeed, it is not only the strength of these connections that matters, but also the pathways through which species contribute to place attachment as people’s connections may differ across species. Understanding this intersection will be important to advance our understanding of human–nature relationships and for informing conservation practice, where species and place are rarely managed in isolation.

**Fig. 1:**
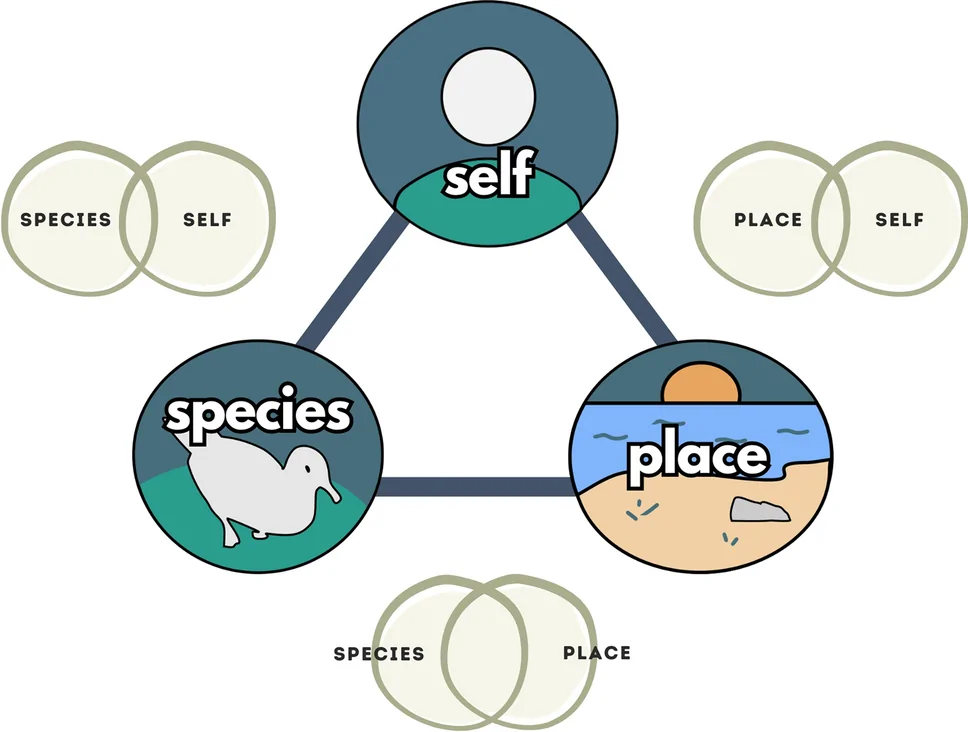
Conceptual framework illustrating the three relational dimensions: Place–Self, Species–Self, and Species–Place. This framework builds on the Inclusion of Self scale to explore the relationships between self, species, and place

To address these gaps, here we examine the ways in which attachments to place and species relate. Specifically, we present findings examining the relationship people have between two different migratory seabirds on an iconic landmark in Tasmania, Australia. By comparing species–place relationships between two species, this study offers a unique perspective on how species can contribute differently to place attachment. To do so, we used a novel adaptation of the Inclusion of Self (IOS) scale that considers Place-Self, Species–Self, and Species–Place, relationships (Fig. 1). To our knowledge, this is the first adaption of the IOS to examine closeness to specific species or places and the interaction between them. We also interviewed members of the community on Bruny Island to identify narrative-based insights into how and why attachment to place and species are formed. Here, the study aimed to address two key questions: (1) What is the relative role of perceived species–self and species–place relationships in people’s attachment to place (i.e. place-self)? and (2) what is the nature of these relationships and how were they formed?

## Materials and methods

To study the triadic self–place–species relationships, we developed a two-stage analytic workflow. First, we sought to quantitatively elicit participant’s relationship with self, place, and species as pairwise relationships. To do so, we extended the IOS framework to species and place. Thus, we adopted an explanatory sequential mixed-methods design (Ivankova et al. 2006) where we first quantitatively assessed participants’ self–species–place relationships. In the second phase, we drew on semi-structured interviews with a subset of respondents to interpret and validate the patterns that emerged from the survey data. We detail each phase below.

### Study context and participants

**Fig. 2:**
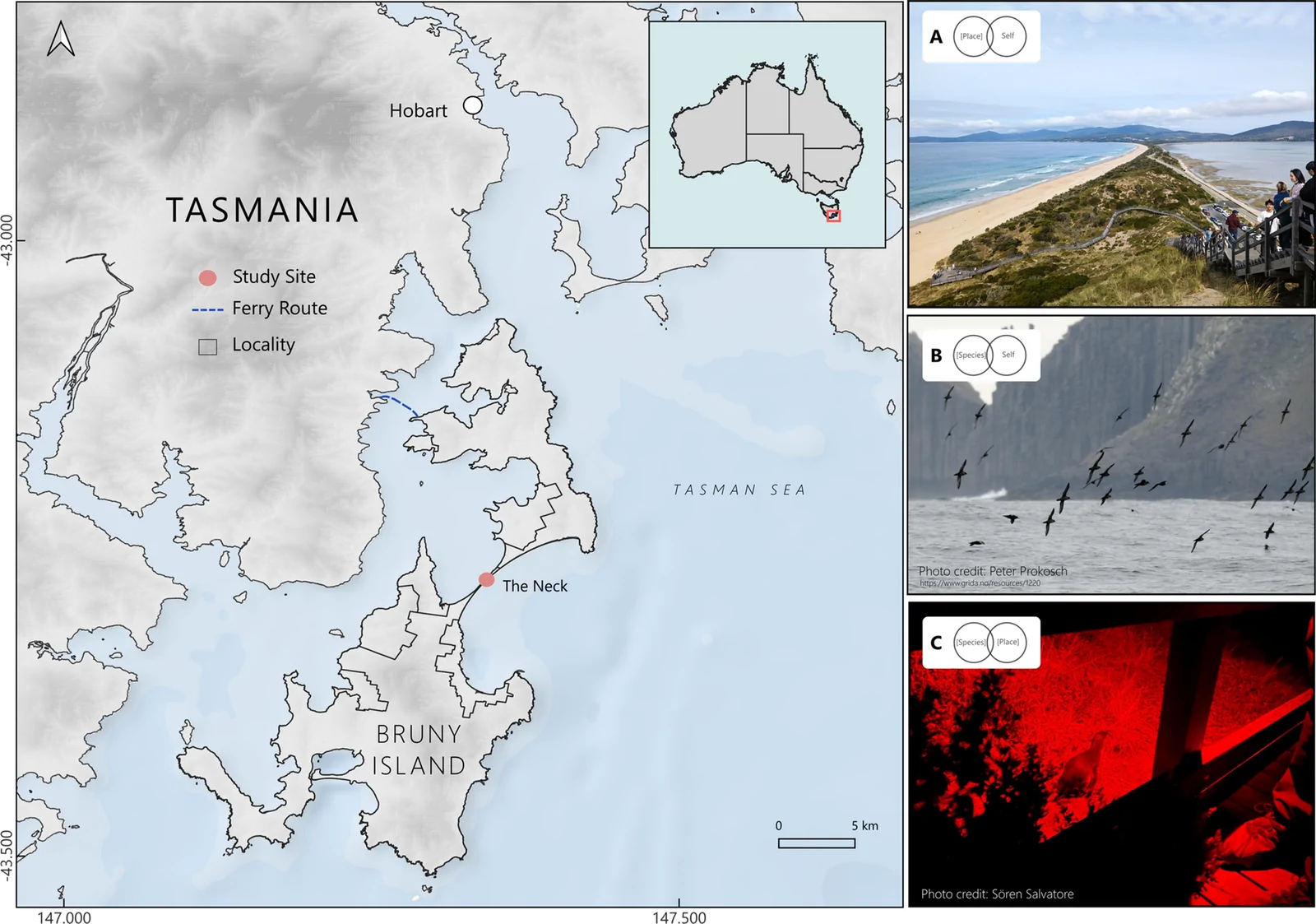
Three scale measures were developed: **A** Place-Self-participants’ relationship to The Neck, **B** Species–Self-participant’s relationship to fairy penguins and muttonbirds, irrespective of place, and **C** Species–Place–participant’s perceived belonging of fairy penguins and muttonbirds to The Neck. The study site (pink), "The Neck", is a narrow isthmus that connects the north and south of Bruny Island, an island off the southeastern coast of Tasmania, Australia

This study took place within the context of people’s connection to fairy penguins (*Eudyptula* minor) and short-tailed shearwaters (hereafter as muttonbirds, *Ardenna tenuirostris*) at The Neck on Bruny Island, an island located off the southeastern coast of Tasmania, Australia (Fig. 2). The Neck is a prominent landmark of the island for both locals and visitors, renowned for its beauty, ecological significance and breeding habitat for fairy penguins and muttonbirds (Jackson 2006; Mills 2020). Both species are native migratory seabirds that typically emerge at dusk. Fairy penguins, however, are more visibly and symbolically present on the island in the form of signage, marketing, and local establishments. In comparison, muttonbirds are less publicised, but hold strong cultural significance due to the island’s Indigenous harvesting history. Despite the prominent representation of penguins on the island, sightings have declined noticeably in recent years, whereas muttonbirds remain seasonally abundant. Even if sightings are rare, both species leave strong sensory cues including their distinctive squawk and sharp smell of burrows. As such, fairy penguins and muttonbirds provide a compelling case for examining how presence, and symbolic or cultural representation can shape distinct pathways in people–species–place relationships.

The survey targeted individuals with a connection to Bruny Island, including permanent residents, and visitors who own a holiday home, have strong family ties, derive a livelihood there, or visit regularly for recreation. As length of residency may also influence place attachment (Raymond et al. 2010a), participants were asked to indicate the length of their connection to the island so that it can to be used as a variable to examine variability within respondent groups. Participants were recruited through three methods: invited participants from a prior survey on species–attachment on Bruny Island, distributed the survey at local community events, and mail-dropped the survey to residents on the island. The procedure was approved by the University of Tasmania Human Research Ethics Committee (HREC29693). A total of 397 surveys were distributed, and 95 were returned for a response rate of 23%. After excluding incomplete responses, 93 responses were retained for analysis. Requests for interviews targeted participants from the prior survey on species attachment. Of the initial 97 interested participants, 28 agreed to semi-structured individual interviews which were conducted between May and September 2024.

### Extension of the inclusion of self scale

The Inclusion of Self Scale was initially developed to capture the cognitive overlap people feel with another entity (Aron et al. 1992). It presents seven increasingly overlapping pair of circles where each pair represents a different degree of overlap. For each scale, participants were asked to draw a rectangle around the pair of circles that best described their relationship to the specified label. The scale ranges from two separate circles (i.e. indicating no perceived connection to place/species, post-scored as 1), to two fully overlapping circles (i.e. indicating one with place/species, post-scored as 7). The Inclusion of Self Scale has been extended to Nature through the introduced “Inclusion of Nature in Self Scale” (Schultz 2002). This scale was also originally modified from the Inclusion of Self scale (Aron et al. 1992) to directly assess inclusion with the natural environment (i.e. how interconnected one is with nature). While this measuring instrument has been found to be reliable (Martin and Czellar 2016), there is, however, not yet a version of the scale for specific places or elements in nature. As a pilot test, we therefore extend the Inclusion of Self Scale (Aron et al. 1992) to concepts of places and species (Place-Self, Species–Self, Species–Place) (Fig. 2; see Appendix S1 for the full survey). To capture the distinct ways participants relate to fairy penguins and muttonbirds at the Neck, five scale items were developed across the three measures. The development of these measures is detailed below.

#### Place in self scale (place-self)

To measure the Inclusion of Place in Self Scale (Place-Self), we adapted the introduced “Inclusion of Nature in Self Scale” (Schultz 2002) to be place specific. Context in nature is important, as place attachment research demonstrates that people form distinct emotional and cognitive bonds with specific settings beyond the physical aspect of human–nature relationships (Nisbet et al. 2009; Gosling and Williams 2010; Beery and Wolf-Watz 2014; Soga and Gaston 2025). Therefore, this place-specific scale also reflects the tripartite person-process-place framework (Scannell and Gifford 2010) by situating place as a local component that draws on direct place-based experiences rather than a general nature backdrop. As a result, participants were asked to express this relationship by drawing a rectangle around one of seven sets of circles that best described their relationship to the Neck.

#### Species in self scale (species-self)

The Inclusion of Species in Self Scale (Species–Self) is a modified scale of the Inclusion of Self (IOS) by Aron et al. (1992). The IOS has been extensively used and proven to be a versatile and reliable tool for assessing ones closeness to interpersonal relationships (i.e. romantic, friendship or family Aron et al. 1992; Gächter et al. 2015). Yet the literature on environmental psychology and human–animal studies demonstrate that people also form distinct, meaningful bonds with specific species that are often difficult to capture in a simple scale. Therefore, this species–specific scale retains the clarity of the original IOS format (Aron et al. 1992) but targets species-level connections to capture people’s deep emotional ties with a focal species (Smith and Sutton 2008; Krasny et al. 2014; Korach et al. 2020). As such Species-Self describes how species become part of how people think about who they are. For each species, an identical scale was applied to measure participants’ relationships with that species. Participants were asked: “Please draw a rectangle around the set of circles that best describes your relationship to (1) fairy penguins, and (2) muttonbirds”.

#### Species in place scale (species–place)

We introduce the Inclusion of Species in Place Scale (Species–Place) to capture the extent to which a species is perceived to belong within a given place. Unlike the Place-Self and Species–Self that measures individuals as highly place attached or species attached, Species–Place emphasises the ecological linkage between the two. It is distinct in that it measures how one’s combined place and species attachment is mediated by ecological knowledge and awareness of how species presence can shape meaning of a place. This scale extends the IOS to reflect the linkage between place and species, but draws on various frameworks and theories. Environmental identity and place identity theories suggests that forming a bond with specific nonhuman elements—such as a specific species within a natural environment, can strengthens one’s psychological sense of self and belonging in that environment (Brook 2003; Clayton 2003; Rollero and De Piccoli 2010; Wilkinson et al. 2014). Drawing on these insights, the perceived connection between a place and its species shapes how people’s cognitive elements come together to form an integrated “Species–Place” relationship that translates how a species belongs to a place both ecologically and experientially. In this context, two scales were developed to measure participants perceived belonging of a specific species to a place. Participants were asked: “Please draw a rectangle around the set of circles that best describes your association between (1) fairy penguins, and (2) muttonbirds, and the Neck”.

### Interviews

The semi-structured interviews were designed to be conversational, using prompts to understand the underlying characteristics of species–people relationships in place attachment and reflected questions examining the roles of fairy penguins and muttonbirds at The Neck, and how attachment to each species differ (see Appendix S2).The interview guide was structured to test a conceptual framework identifying key roles of species in place attachment (Shum et al. 2025). In the context of place, these roles include: (1)* identification-* how participants label species*, *(2)* association-* how participants reference and relate to species*, *(3)* interaction-* how people perceive the present species*, *(4)* knowledge-* the extent and understanding of species*,* and (5)* kinship-* the relational connection to species (Shum et al. 2025). Interviews were conducted in English, primarily in person, with Zoom or telephone used as alternatives when needed. Sessions typically lasted from 20 to 40 min, and all participants consented to be recorded using an audio recorder application.

### Data analysis

To test whether species–self and species–place can predict place-self relationships, data was first analysed by examining the distribution of each IOS-derived scale. Normality was assessed using Shapiro–Wilk tests, which indicated Species–Self, and Species–Place were non-normally distributed. A Wilcoxon signed-rank test was therefore used to compare Species–Self and Species–Place scores between the two species. As these were the variables of interest in comparing scores between the two species, Place-Self scores were not subject to this test. Next, we calculated Pearson correlations to determine the strength, direction, and overlap among Place-Self, Species–Self, and Species–Place, as well as measures for internal consistency across the two species. A multiple linear regression analysis was also carried out separately for each species to predict Place-Self measures from Species–Place and its corresponding Species–Self score. Length of residency was also tested as an explanatory variable in the analysis to account for any differences that may be associated with positionality in place.

For the qualitative data, participant interviews were coded in NVIVO, using an abductive approach (Awuzie and McDermott 2017). First, we coded all references to fairy penguins and muttonbirds separately, against five out of the six key roles of species in place attachment described above (Shum et al. 2025): (1)* identification, *(2)* association, *(3) *interaction, *(4)* knowledge, *(5) *kinship*. Drawing on the codes derived from the five key roles, we then qualitatively explored how these varied across respondents with differing lengths of residency. This reflects the sixth key role *actors,* which describes how societal position shapes species–place relationships. Qualitative responses were compared between short-term residents (less than 10 years), and long-term residents (more than 10 years) to assess how length of residency shapes expressed pathways. We also coded comments detailing species–specific preferences. In the second round of coding, we refined and expanded these categories to identify the underlying characteristics of self-species–place relationships. Throughout this process, both co-authors cross-checked a random subset of coded interviews to ensure consistency.

## Results

### Descriptive statistics

A total of 93 individuals (57 females, 34 males, 2 other) completed the questionnaire, with an average age of 51 (range 20–85 years). The majority of participants lived on the island permanently (*n* = 69), followed by for recreational or work purposes (*n* = 13), as a holiday home (*n* = 9), or for other means (*n* = 2). On average, participants have spent more than 10 years living on the island (*n* = 59). Of the 93 questionnaire respondents, 28 individuals also agreed to be interviewed. Within this group of interviewees, length of residency was evenly distributed, with 14 individuals reporting a connection of less than 10 years and the remaining 14 indicating 10 years or more.

### Species-self, place-self, and species–place relationships

**Fig. 3:**
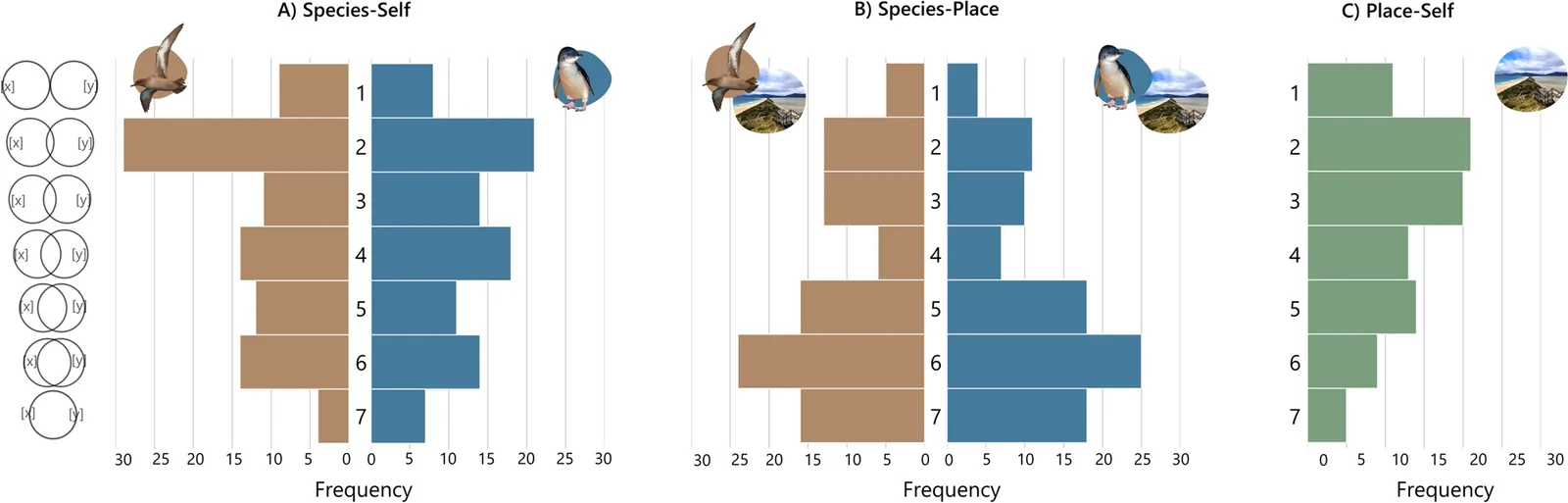
Distribution of overlap scores (1–7) adapted from the Inclusion of Self Scale: **A** Inclusion of Species in Self (Species–Self), **B** Inclusion of Species in Place (Species–Place), and **C** Inclusion of Place in Self (Place-Self). Each pair of circles represents their perceived connection to The Neck, fairy penguins, or muttonbirds (x, y) where 1 indicates no perceived connection, and 7 indicates complete overlap with place and/or species

Between the two species, we found significant differences between species for both Species–Self (*V* = 582, *p* = 0.047), and Species–Place (*V* = 379, *p* = 0.007) scores. When considering the distribution of each IOS-derived scale, we found that apart from Species–Place, Species-Self and Place-Self measures leaned towards a weaker connection rather than the extremes of no overlap or complete overlap (Fig. 3). In particular, for both fairy penguins and muttonbirds, the Species–Place scores peak at 6 out of 7 in terms of perceived connection and overlap, with 25 and 24 respondents, respectively, selecting that overlap value, suggesting that most people perceive these species with The Neck. In contrast, Species–Self measures show more variation between the two species with participants generally feeling less connected with muttonbirds than fairy penguins. For fairy penguins, people’s connection was spread between a low connection score of 2 (*n* = 21), a neutral score of 4 (*n* = 18) and a high overlap score of 6 (*n* = 14). For muttonbirds, most participants selected a score of 2 (*n* = 29). However, scores for the strongest overlap were similar between fairy penguins and muttonbirds with 7 and 6 participants selecting a score of 7, respectively. Lastly, people’s connection to The Neck (Place-Self) appears to be low with participant generally selecting a value of 2 (*n* = 21), and 3 (*n* = 20).

**Fig. 4:**
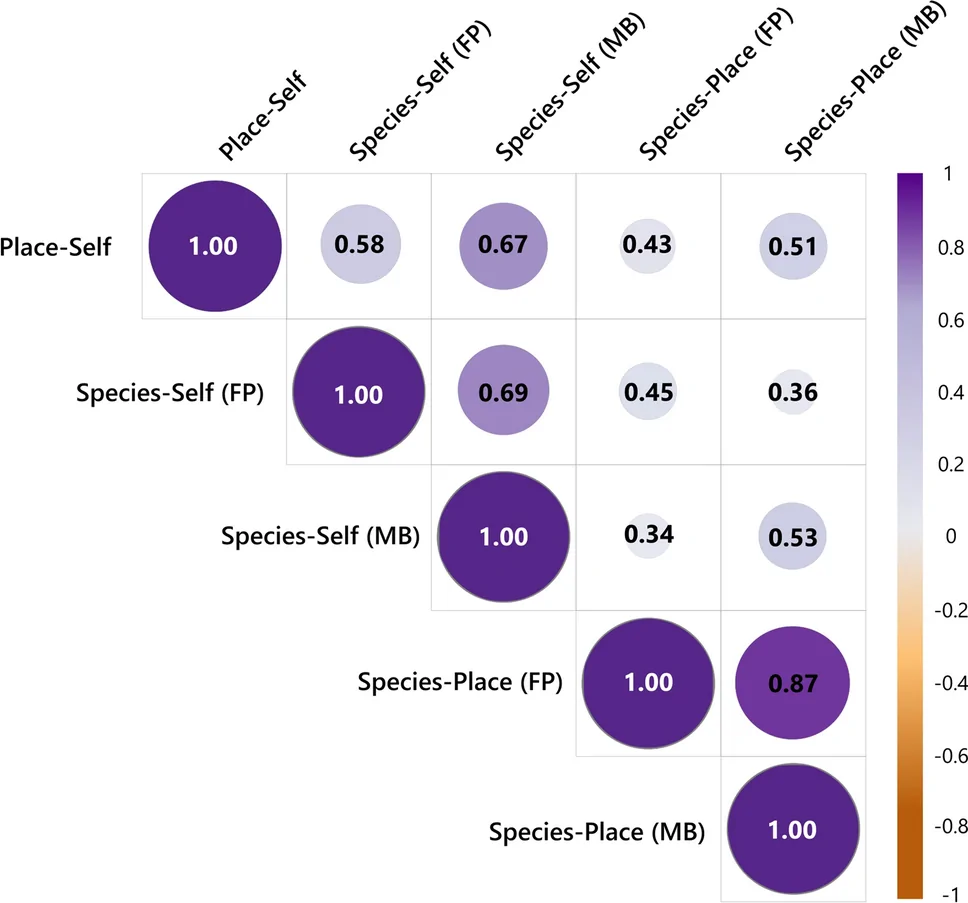
Correlation matrix plot of Place-Self, Species–Self, and Species–Place measures of both species (fairy penguins (FP), muttonbirds (MB)). Circle size and colour intensity represent the strength of Pearson correlation coefficients

A Pearson correlation analysis showed that all scale measures were positively correlated and statistically significant (*p* < 0.001) (Fig. 4, Table 1). Internal consistency was demonstrated across both species within Species–Self and Species–Place scales, meaning that participants who tend to have strong connections with fairy penguins also tend to rate themselves similarly with muttonbirds. In the same manner, participants who see fairy penguins as part of The Neck also associate muttonbirds in a similar way (*r* = 0.87). Correlation between Species-Self and Species–Place reveals a moderate relationship with muttonbirds scoring higher than fairy penguins (*r* = 0.53, 0.45, respectively). The same pattern was observed between Species–Place and Place-Self with muttonbirds (*r* = 0.51), and fairy penguins (*r* = 0.43). For Species-Self and Place-Self scales, a strong correlation was observed for both fairy penguins (*r* = 0.58) and muttonbirds (0.67). These correlations suggests that both Place-Self and Species-Self measures are positively associated with participants’ perceived Species–Place connection.

**Table 1:**
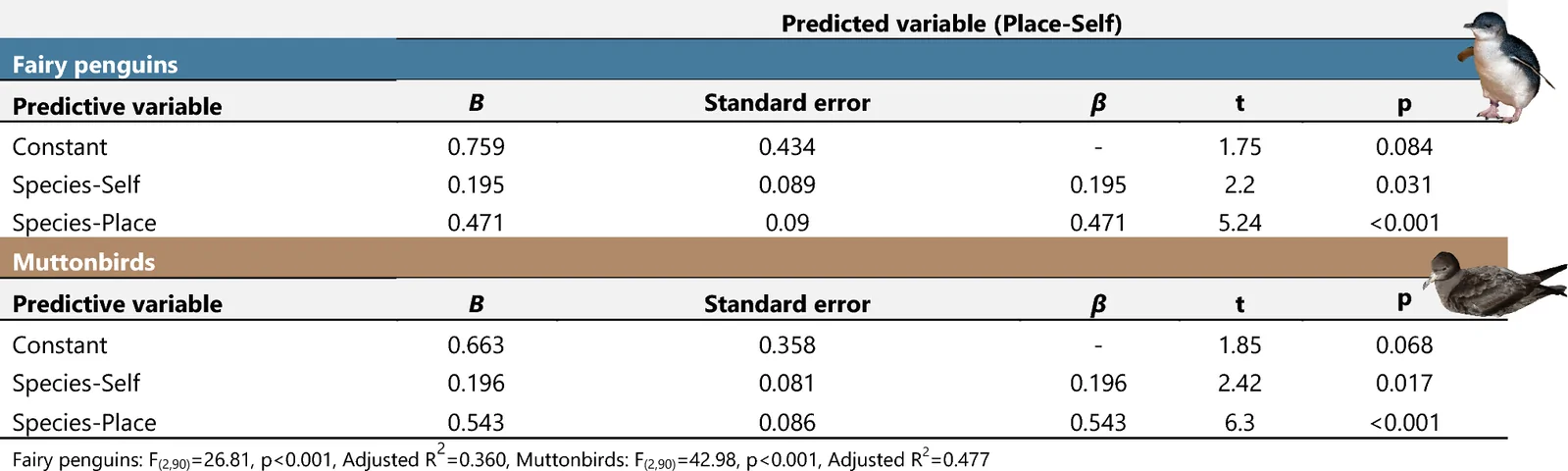
Results of a multiple linear regression analysis between fairy penguins and muttonbirds on adapted inclusion of self-scales (Species–Self, and Species–Place) as the predictor of Place-Self

### Role of species–self and species–place relationships to place attachment

Regression models for fairy penguins and muttonbirds both revealed Species–Place and Species–Self contribute independently to predicting Place-Self relationships (Table 1). For fairy penguins, the overall model was statistically significant, F(2,90) = 26.81, *p* < 0.001, and explained 37.3% of the variance in Place-Self (adjusted *R*^2^ = 0.360), with Species–Place having the strongest effect in both models. The results showed that for fairy penguins, Species–Self and Species–Place were significant positive predictors of Place-Self (*β* = 0.195, SE = 0.089, *t*(90) = 2.20, *p* = 0.031, *β* = 0.471, SE = 0.090, *t*(90) = 5.24, *p* < 0.001, respectively). The model for muttonbirds was comparable with the overall model statistically significant, F(2, 90) = 42.98, *p* < 0.001, accounting for 48.9% of the variance in Place-Self (adjusted *R*^2^ = 0.477). Both Species-Place (*β* = 0.543, SE = 0.086, *t*(90) = 6.30, *p* < 0.001) and Species-Self (*β* = 0.196, SE = 0.081, *t*(90) = 2.42, *p* = 0.017) were significant positive predictors of Place-Self. Length of residency did not significantly influence self–species–place relationships.

### Nature of self-species-place relationships

Our quantitative measure of self-species-place relationships revealed that although participants’ connections to different species varied, Species-Place relationships were the strongest predictor to Place-Self relationships. To explore the species-specific pathways underlying this pathway, we asked the 28 participants who took part in follow-up interviews to reflect on the five key roles that species play in fostering place attachment, with the sixth role, *actors*, examined subsequently (Table 2). We found that while participants generally reported lower average scores for Species–Self and Self–Place, the consistently higher Species–Place scores point to distinct, species-specific pathway which we outline below.

**Table 2:**
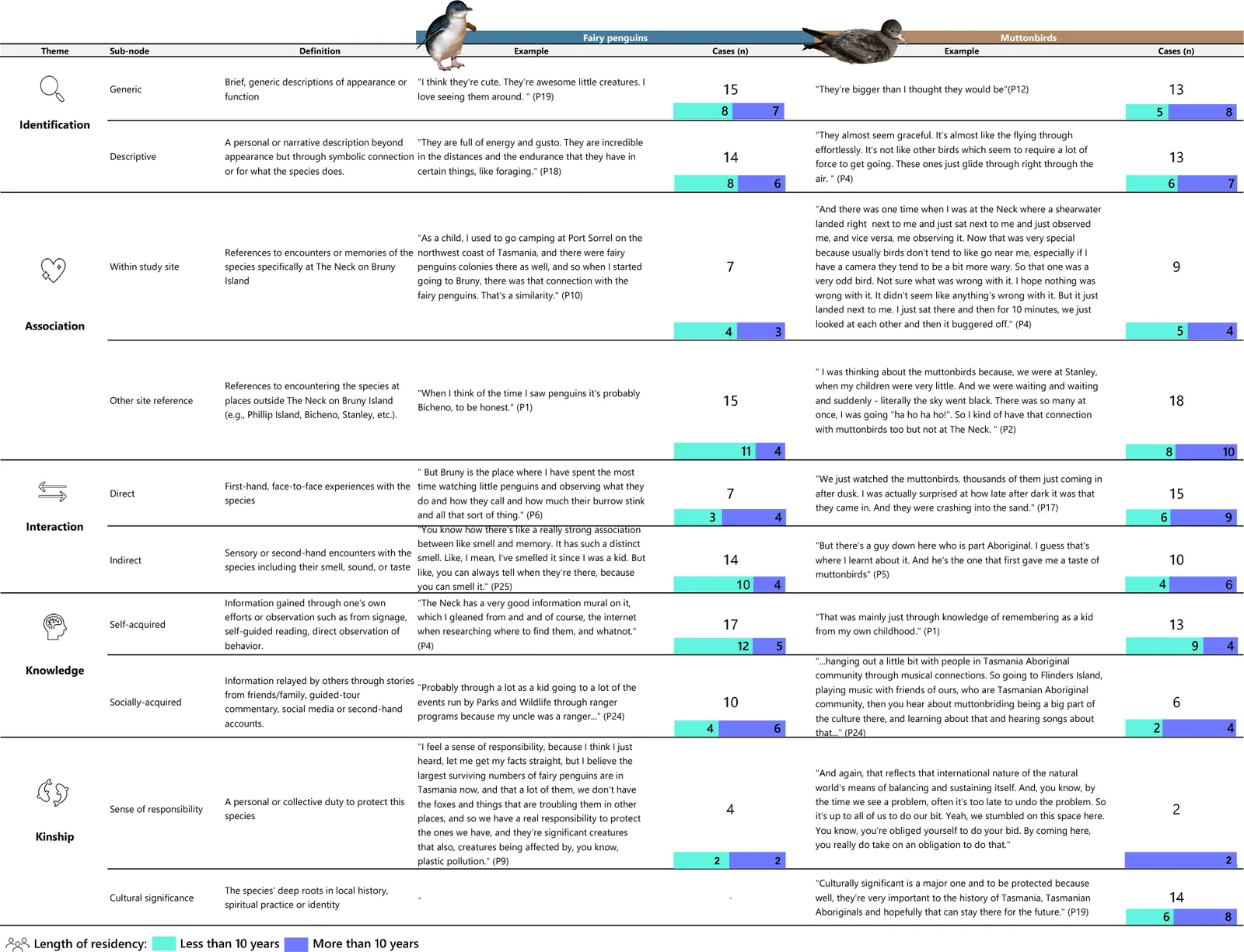
Themes and sub-nodes derived from interviews comparing fairy penguins and muttonbirds. Case counts (*n*) indicate how many participants referenced each sub-node. Counts are further disaggregated by actors (length of residency), with coloured bars distinguishing participants residing less than 10 years (light blue) and more than 10 years (dark blue)

A recurring theme for both species was their association with sites beyond The Neck. For many participants, encounters with fairy penguins or muttonbirds in other places appeared to help draw their connection out to The Neck as well. As one participant explained: “*As a child, I used to go camping at Port Sorell on the northwest coast of Tasmania, and there were fairy penguin colonies there as well, and so when I started going to Bruny, there was that connection with the fairy penguins. That’s a similarity*” (P10).

The lack of direct association of species to The Neck might suggest that direct encounters (i.e. seeing or touching) would be more valued than indirect exposures. However, this was not supported by our findings. Although more participants described direct interactions with muttonbirds, fairy penguins were generally regarded more positively through indirect encounters. For example, participants frequently referred to the penguins’ “*distinct smell*” (P25) and noted that they were more often heard than seen. One participant reflected that even without a sighting, the shared anticipation created a meaningful experience: “*We didn’t actually see any penguins or any muttonbirds that night, but the excitement of people, that was really lovely, that they thought they might get to see one” (P10)”.* Even when penguins were observed, participants often described their behaviour more than its physical traits: "*They’re more present. They’re on the ground. The muttonbirds are in the air mostly*" (P14), and "*Just the way they move… the way they jump. They seem to be affectionate within their own family groups*" (P22).

Knowledge about the species also appeared to shape participants’ connections, with many demonstrating greater familiarity with fairy penguins than with muttonbirds. This knowledge was often acquired more independently but also through social channels. As one participant noted, “*There’s the visitor’s centre and there’s always more information about them (fairy penguins). And they’re something that maybe you learn about more, whereas shearwater I’d never heard of before I saw them the first time. And then even since then, they don’t get much press, I guess, apart from [the] occasional*” (P11).

For some participants, however, it was the distinctive ecological role and behavioural complexity of the muttonbirds that fostered a deeper appreciation: "*There’s more complexity in their lives and the complexity of their lives is affected more by the climate change issues because it’s planet wide*" (P20). Compared to others, being able to see fairy penguins in a more predictable manner was more important: "*…the fairy penguins […] they're much easier to see. You know, like when we're at the neck, you see muttonbirds flying in but then they went into their burrows, they flew in you didn't really see them. Whereas with the penguins, you can watch them coming up out of the water. And going all the way up to their burrows so you get a much more intense experience watching them and then with kids, the penguins, who can resist them! (P22).*”

This is perhaps an indication of the unique way that fairy penguins and muttonbirds complement one another—both as native and co-occurring species of the site. While fairy penguins often evoke more accessible and visible encounters, muttonbirds are strongly tied to The Neck through a more culturally mediated, and ecological connection. Coupled with the convergence of these two species at an iconic landmark, this combination forms a defining characteristic of the place. As one participant described, "*I think the meeting of these two species in that one stretch of habitat is magnificent. That is such a boasting point for that place, that’s precious*" (P9).

In exploring participant’s self-species-place relationships, all five themes were distinctly represented between species, with the exception of *kinship*, which was expressed primarily in relation to muttonbirds. These relationships echo the role of muttonbirds as a culturally significant species, though perhaps frequently perceived as collective or symbolic, rather than personal. As one participant reflected, "*I suppose for me, it’s more about the cultural significance of the shearwater rather than any personal attachment to the bird*" (P10). These narratives suggest that among *identification, association, interaction, knowledge, and kinship,* the latter is more likely to be shaped by collective cultural values than by personal experience.

Disaggregating the five key roles by actors revealed a clear residency effect where shorter-term residents showed some distinct pathways for fairy penguins than long-term residents. Short-term residents appear to be more likely to reference other sites when discussing penguins (*n* = 11, 73%), and to reference indirect encounters (*n* = 10, 71%). Although residency effects were weaker for muttonbirds overall, independently acquired knowledge was more common among short-term residents for both species (fairy penguins: *n* = 12, 71%, muttonbirds: *n* = 9. 69%). In contrast, long-term residents (more than 10 years) were more likely to report knowledge gained through social channels.

## Discussion

This study examines the distinct connections people form with species and place using a triadic approach to eliciting and understanding the self-species-place relationship. By exploring relationships of self to both the species and the places they inhabit, our results revealed three key findings: (1) both Species-Self and Species-Place relationships were significantly related to Place-Self (i.e. place attachment), but respondents’ understanding of species in place had the stronger relationship; (2) relating to Species-Place relationships, the interviews revealed species-specific pathways for acquiring knowledge and interacting with each species; and (3) Self-Species-Place relationships support the six key roles of species as placemakers, with all roles frequently expressed except *kinship* which emerged distinctively for muttonbirds.

### The role of species-self and species-place relationships

Our findings demonstrate that Species-Self and (to a greater extent) Species-Place relationships were significantly related to place attachment. This aligns with concepts related to connectedness to nature or the “noticing nature cycle”, in which knowledge is a precursor to noticing, engagement, and the formation of deeper connections with nature (Restall and Conrad 2015; Hamlin and Richardson 2022; Garfinkel et al. 2024). The act of noticing nature triggers a cycle of engagement and connection because it encourages people to reflect on how species and place belong together (Richardson et al. 2022). As Richardson et al. (2022) demonstrate, activities such as watching wildlife, listening to birdsong, or photographing species explain greater variation in nature connectedness than merely time spent in nature. In our study, this cycle was evident even with contrasting Place-Self and Species-Self scores. Supported by the qualitative responses, species are revealed to be an essential component to the meaning of place. Participants described key moments of noticing (e.g. sensing a species’ presence), interaction (guided tours, sensory cues), and connection built through cultural narratives and ecological knowledge, which we explore in greater detail below.

### Species specific pathways in species-place relationships

The consistent differences in how fairy penguins and muttonbirds engage people is particularly relevant for understanding how species-place relationships are derived. Prior research has shown that species identified as “charismatic” or “iconic” often hold symbolic power, engaging wider audiences in ways that extend beyond their immediate ecological role (Ducarme et al. 2013). Reflected in the stronger reported Species–Self relationships, fairy penguins embodied greater charismatic appeal, with participants highlighting how their visibility, predictable behaviour, and ease of encounter made interactions with the species more accessible. Similar patterns have been observed for other charismatic species such as dolphins (Smith et al. 2024), and sea turtles (Campbell and Smith 2006), where visibility and predictability enhance emotional connection.

Notably, however, participants reported few direct interactions with penguins, suggesting that the influence of charisma extends beyond physical encounters and instead may strengthen through other indirect measures. For some participants, the distinct smell of penguins demonstrates the unique role of smell in Species-Place relationships as a meaningful pathway of connection (Press and Minta 2000). This emphasises that even if a species is not universally charismatic (i.e. muttonbirds), other cues related to the species can still be used to help define a place’s identity. This is an important finding, as it explains how distinct cues such as sound or smell can create important pathways to build relationships. Therefore, the way people connect with species in place is unique and highly relational and context specific. It suggests that conservation, tourism, and land-use decision-making should move beyond conventional visual appeal to incorporate the sensory and relational dimensions into future management to resonate with how people actually experience and value species within place.

One of the pathways we identified highlights the importance of knowledge in shaping self-species-place relationships. As mentioned previously, the noticing nature cycle emphasises noticing as a pathway to nature connectedness, whereby attention to nature creates a motivational pull to learn, observe, and engage more deeply through self-led cycles of exposure and reflection (Lengieza and Swim 2021; Pocock et al. 2023). Noticing however, is not only internally driven—it can also be leveraged externally in different situational contexts such as guided walks, storytelling, or shared social learning. In this way, knowledge can be introduced through educational campaigns such as interpretive signage (Ballantyne et al. 2011; Higgins et al. 2015), which can draw attention to species that may otherwise be difficult to observe, such as nocturnal or migratory species. The intent of additional signage is not to assume that filling a knowledge deficit will strengthen attachment or action, but rather to provide context and recognition for less visible species, thereby creating opportunities for awareness and engagement. These opportunities can be further reinforced through personal memories formed during encounters with the species (Buckley 2022), which in turn may contribute to the development of pathways towards stronger species-place relationship.

For highly charismatic species like fairy penguins, knowledge was often more readily accessible through social channels, such as guided tours or through family and friends. By contrast, for species less prominently associated with a place, our findings suggest that participants who learned more about the species’ unique ecological role fostered a stronger connection to place. In these cases, the added layer of complexity (i.e. understanding the species’ migration patterns, or broader ecosystem processes), provided a distinct pathway for strengthening the relationship. This finding suggests that practitioners can present opportunities to broaden public knowledge of species, including the integration of Indigenous knowledge systems to support intercultural management approaches as a pathway for attachment building (Burgos-Ayala et al. 2020). Building knowledge, such as through educational signage, is a common practice already used by practitioners but designed to address knowledge deficits whereas our findings suggest these practices could shift to focus on knowledge as a means of attachment building. This mirrors findings in other contexts where ecological interpretation and education have strengthened people’s attachment to species by fostering species-focused recreation like birdwatching (Connell 2009), and linking them to broader environmental narratives and conservation concerns (Bowen-Jones and Entwistle 2002).

### Pathways of self-species-place relationships

Our narrative-based insights reveal that people’s relationships with species and place can be expressed through six main pathways that shape how they experience species within place. These pathways are formed through personal, cultural, ecological, and social dimensions that include *identification, association, interaction, knowledge, kinship, and actors*.

Although kinship was the least frequently expressed role, its distinct presence in relation to muttonbirds is an important consideration. That is, *kinship* reflected a deeply relational form of connection that extended beyond familiarity or appreciation. For many participants, *kinship* emerged as a collective form of connection, grounded in shared cultural meanings over personal attachment. This pattern is consistent with research on “cultural keystone species”, a term used to describe species that shape the cultural identity of a community or place through their symbolic and practical significance (Garibaldi and Turner 2004). Such communal attributes suggest that self-species-place relationships can persist even among individuals who do not have direct or frequent personal encounters. For conservationists and practitioners, this moves beyond approaches centred on fostering individual attachment and instead, presents opportunities to design engagement strategies that encourage storytelling, scenario planning, and education to reinforce shared identities and collective stewardship ( Raymond et al. 2010b; Levstek et al. 2024; Galang et al. 2025).

For non-charismatic species, our findings support muttonbirds as a tangible link to The Neck’s natural heritage. In other words, even visitors who don’t form a personal bond with the birds come to associate The Neck with the presence of these migratory birds. This supports the recent call to align cultural values with local priorities through the proposed metric of “biocultural status” (Reyes-García et al. 2023), and reinforces the notion that even “non-charismatic” species can still function as powerful placemakers by shaping the character and meaning of a location through cultural and symbolic connections (Forristal et al. 2014). These findings demonstrate the value of framing species (where relevant) through a kinship-based appeal as it represents a stronger and more enduring form of connection where it does not only rely on personal attachment but also persists through knowledge of social channels and cultural significance.

When put together, participants consistently linked their experience of place to the presence of wildlife—echoing the concept of species as placemakers (Shum et al. 2025). Interestingly, participants often referred to fairy penguins and muttonbirds in relation to other sites, suggesting that indirect associations also bind these species to The Neck. This reflects a form of duality between the two species (Hussain 2019): the charismatic appeal of fairy penguins contrasted with the strong ecological and cultural association of muttonbirds. Together, the two species contribute complementary qualities to The Neck’s identity—described by participants as the “precious” convergence of species. These findings suggest that different pathways of connection operate simultaneously, where species-place relationships are both ecologically grounded and culturally mediated. In other words, complementary species that embody different values may have an additive effect in strengthening species-place relationships. Conservation messages and actions that link multiple species to a place simultaneously may therefore draw on the distinct values associated with each species and broaden the range of relational pathways through which people connect to place. By doing so, diverse meanings and forms of attachment can coexist and reinforce one another.

The influence of residency effect (i.e. *actors,* separated here based on time they’ve lived on island as a key differentiating attribute) on the pathways through which species-place relationships are formed underscores the value of adapting messaging approaches to different audience groups. For short-term residents, the number of references to other sites and indirect encounters suggest that place meanings may be built more through broader regional comparisons rather than place-specific familiarity. This pattern is consistent with early-stage place attachment where meanings are built from prior experiences and external reference points rather than long-term immersion (Wilkinson et al. 2014; Di Masso et al. 2019; Man Cheng et al. 2022). Communicating strategies should therefore aim to draw parallels to similar species and sites encountered elsewhere in-situ. For example, signage illustrating where fairy penguins and muttonbirds can also be seen in Tasmania or in other parts of Australia can help people link new places to existing frames of reference. In contrast, for long-term residents, whose knowledge is more socially embedded, messaging that supports shared storytelling and community gatherings may better reinforce existing attachments by allowing residents to collectively reflect on a place (Son and Krolikowski 2025).

### Future directions and limitations

To our knowledge, this is first study to extend the Inclusion of Self Scale to examine specific relationships between people, species, and place, and how these dimensions integrate. While this opens several avenues for further research on measuring species–place relationships, its exploratory nature also presents some caveats. First, the Species-Self, Place-Self and Species-Place scales developed here highlights the potential of a triadic approach but will require more rigorous testing to establish its validity and reliability. For example, the spatial scale of the study may also explain lower levels of Place-Self since people may feel more strongly connected to the island as a whole than to a specific site such as The Neck. Lewicka (2010) argues that place attachment can be better explained at some spatial scales than others, with weaker predictive power if place-specific variables are misaligned with the scale of the analysis. This is especially relevant when Species-Self is less constrained by site boundaries and can translate across broader spatial scales. More research is required to acknowledge these differences and to examine how Species-Self connections may compensate for site-specific place attachment across scales.

Another promising direction is the use of an illustrative approach to the IOS scale, such as that used by Kleespies et al. (2021), which has been shown to help individuals with lower levels of imagination or abstraction more accurately reflect their connection to nature. Incorporating such visuals would also enable more reliable comparisons across scale measures, as we observed anecdotally that participants responded more confidently when the tasks was accompanied by a visual or personalised example.

Additionally, as a case study focused on a unique set of species and a specific place, generalisations to other regions, species, or cultural contexts should be made with caution. We note that by asking participants to reflect directly on the five key placemaking roles may introduce a degree of circularity in the findings. Further validation could be achieved by incorporating control assessments that explore people’s relationships with place without explicit reference to the species, as well as comparative studies (Martin and Czellar 2016) examining the same species across different sites. This is especially relevant since our findings suggests that attachment to a species can be transferable between places with similar characteristics. In this context, future work should examine which specific attributes of species-place relationships—*identification, association, interaction, knowledge, kinship, actors*—are transferable and which are more bound to a particular place.

Similarly, because species–place relationships can vary across both species and place attributes, further research is needed to examine how species-specific traits interact with socio-demographic factors such as age, gender, culture, upbringing, profession, and length of residence. For example, unstructured nature experiences during childhood can play a formative role in shaping meaningful connectedness with nature that stretches into adulthood (van Heel et al. 2023). This also includes positionality in place such as between visitors and residents, or between long-term locals and newcomers, different connections and concerns can develop (Lavy and Zavar 2023). In this study, participants were primarily residents and older in age and thus may have already held stronger knowledge and deeper connecting attributes than the general population.

Lastly, while the mixed-methods design allowed us to triangulate statistical patterns with narrative-based insights, the modest sample size may have limited the detection of finer-scale differences between species in understanding species-place relationships. Although length of residency did not significantly influence self–species–place relationships, several interviewees expressed concern that increased tourism, development, and other human–driven disturbances were contributing to a growing sense of disconnection from The Neck. As these anthropogenic pressures continue to intensify, long-term residents may experience negative emotional responses that can manifest oppositional behaviours, such as willingness to collaborate in tourism planning initiatives intended to support more inclusive and considerate forms of change (Lalicic and Garaus 2022). These observations raise important questions about how motives for place visitation and the nature of people’s connections to place may shift under such pressures.

## Conclusion

This study advances understanding of how people connect to places by considering both the strength and nature of human connections to species and place. Notably, species-place relationships emerged as the strongest predictor of place attachment. This underscores the importance of recognising species as an integral part to how people experience and value landscapes. However, there are species-specific pathways to consider when accounting for the diverse ways people come to know and care for wildlife. Our findings support six such pathways: *identification, association, interaction, knowledge, kinship,* and *actors*. Although *kinship* was the least frequently expressed, it remains an important pathway for culturally significant species like muttonbirds. This is particularly emphasised when species contribute to placemaking in ways that are both ecologically grounded and culturally mediated.

For practitioners and policymakers, these insights suggest that conservation planning, visitor management, and place-based engagement efforts should integrate species-specific qualities into decision-making. Enhancing opportunities for noticing and learning—particularly for less visible or culturally important species, may help support more inclusive forms of stewardship. This may take the form of more targeted and meaningful interpretive signage that can broaden ecological and cultural knowledge while encouraging more reflective engagement, thereby reinforcing the pathways through which species-place relationships develop. Likewise, approaches that recognise and protect the cultural and symbolic significance of species can strengthen community attachment and resilience in the face of environmental change. By recognising the interconnected nature of people–species–place relationships, this study encourages strategies that align ecological goals with the ways people experience, value, and connect with the places they care about.

## Supplementary Information

Below is the link to the electronic supplementary material.

Supplementary file 1 (PDF 6982 KB)

Supplementary file 2 (PDF 66 KB)

## Data Availability

The anonymised data collected in this study are publicly available through the University of Tasmania Research Data Portal (RDP): online research repository: https://doi.org/10.25959/1she-sa45.
