# Supplementary material for: Characterising people–species–place relationships
Source: Ambio. 2026 May 23;55(12):2905–20. doi: 10.1007/s13280-026-02411-0 (PMC13731557; doi:10.1007/s13280-026-02411-0)
Supplement: Supplementary file 1 [file 13280_2026_2411_MOESM1_ESM.pdf]

Supplementary Information: This Supplementary Information has not been peer reviewed.

Title: Characterising people-species-place relationships

Appendix S1: The following is the full survey used to explore participant's connection to fairy penguins (*Eudyptula minor*) and short-tailed shearwaters (*Ardenna tenuirostris*) at The Neck on Bruny Island. Note that this is a full survey and not all questions were included in the analysis reported in this paper. The findings presented here draw on the items detailed from page 14 onwards.

ESA

UNIVERSITY of TASMANIA

TASMANIAN LAND CONSERVANCY

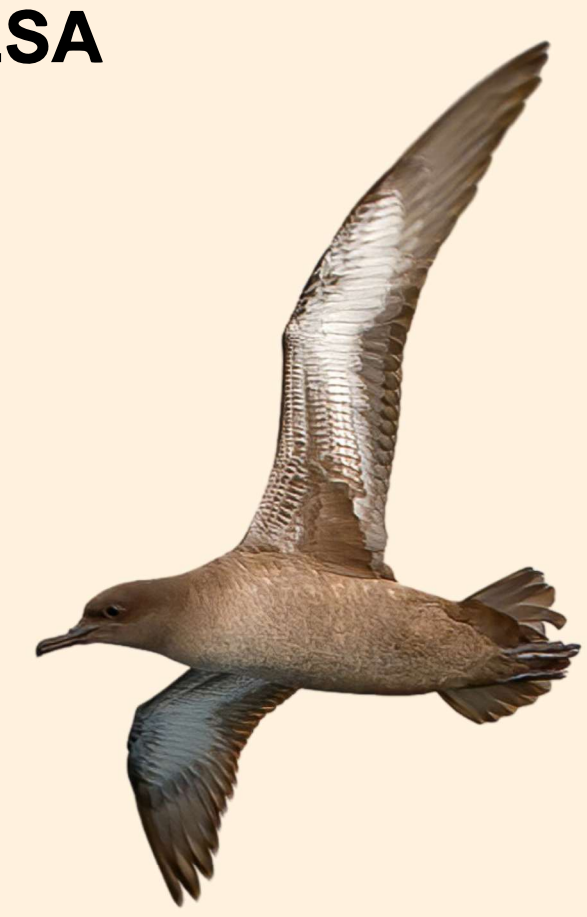

A RESEARCH PROJECT

Connection to Species on Bruny Island

BRUNY ISLAND CHEESE CO.

share your thoughts, take our survey

WIN $100 AT THE BRUNY ISLAND CHEESE CO.

LEARN MORE

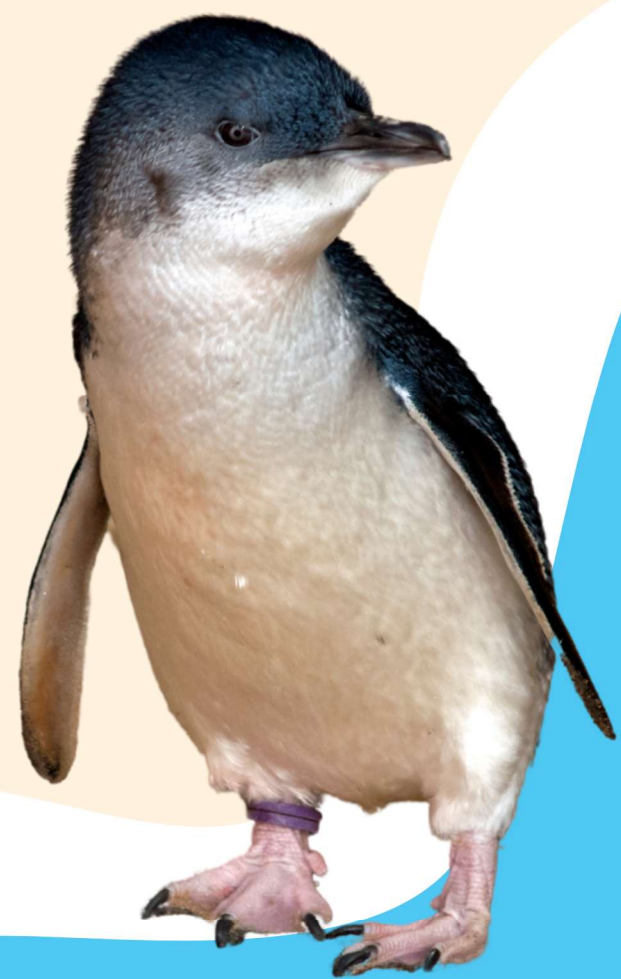

Information Sheet

The Role of Species on your Connection to Place

Thank you for your interest and time! Below, you'll find some technical details about this study. Keep scrolling to find the summarised consent statement to continue, and if you'd like to save this information, there's a download button for your convenience.

Project Overview and Research Purpose

This part of the study seeks to evaluate how valued species on Bruny Island influence the connection that individuals have with specific places on the island. In this case, focus on these relationships will be directed towards fairy penguins (*Eudyptula minor*) and muttonbirds (*Ardenna tenuirostris)* at The Neck. Information will be collected though a questionnaire that will ask about your connection to Bruny Island, and the Neck in more detail, as well as your relationship between valued species and places on the island. This information marks the second phase of a project that seeks to explore how valued species play a role in how we connect to a place.

At the end of the survey, we will ask if you would be interested in participating in the last phase of this project. If you agree to be involved, we will include you in our de-identified dataset so that we may contact you for the next round of the project. Ultimately, the project aims to inform environmental management and policy development about the species and places on Bruny Island that the community values the most.

What Does Participation Involve?

Participation in this study will involve completion of this survey booklet that will take approximately 20-30 minutes minutes of your time. The survey will divided into three sections: 1) determine your level of connection to Bruny Island and the Neck specifically, 2) your relationship with fairy penguins and muttonbirds in relation to the Neck, and 3) demographic information.

Once you've completed the survey, please place it in the prepaid envelope provided and drop it off at any post office. Responses received will be recorded.

Project Team

This project is a component of a PhD thesis being conducted by Edith Shum from the School of Geography and Spatial Sciences at the University of Tasmania (UTAS). The project team consists of Edith Shum and her two academic supervisors, Dr. Vanessa Adams (School of Geography and Spatial Sciences, UTAS), and Dr. Georgina Gurney (School of Geography and Spatial Sciences, UTAS, College of Arts, Society and Education, JCU).

Participation and Withdrawal

Participation in this study is completely voluntary. Should you agree to participate, you may choose to withdraw from the survey at any point by discarding the survey booklet. If you wish to withdraw your responses after they have been mailed, you may do so by sending us an e-mail (provided below) and provide your unique ID. Your unique ID will be available at the top of the of the <Consent> page (pg.4).

Benefits and Risks

Apart from dedicating your time, there are no anticipated risks associated with participating in this study.

While we do not expect direct benefits for participants in this study, this study will advance our knowledge of local values on Bruny Island and how species on Bruny Island can drive or constrain environmental action. Your responses can help shape regional environmental efforts, and thus enhance the socioeconomic well-being of the local community.

By participating in this survey, you may provide your email address to be entered in a draw to win a $100 digital gift card to Bruny Island Cheese Co. In order to be entered in the draw, please provide us with your e-mail address when prompted. Your e-mail will only be accessible to the research team and will only be used to provide you with your voucher should you win the draw.

Confidentiality

All information you provide will be treated confidentially and will only be accessible to the research team. In reporting the results of this research, no specific participant will be attributed to any statement or finding. Your e-mail address (should you provide it), residential information and any other personal information you provide will not be included in any publications or other outputs resulting from this study. All data collected in this study will be coded in a non-identifiable manner and subsequently analysed and reported in such a way that responses cannot to be linked to any individuals.

Responses will be stored in a secure database that contains every response we receive (located in the Geography and Spatial Sciences building, Sandy Bay Campus, UTAS). De-identified data will be archived via the University of Tasmania Research Data Portal.

How will my information be used?

It is anticipated that the de-identified data (where you opt into providing contact details for our subsequent follow up contacts) obtained through this survey will be used to guide the final stage of this project, including a semi-structured interview or focus group workshop that will help capture the changing nature of the relationship between species, and places. The analysed survey data will be used strictly for research purposes and may be published and/or presented in a variety of forums. This includes journal publications and conference presentations. This information will also be submitted to the University of Tasmania in mid-2026 as part of Edith Shum's PhD thesis.

Future surveys

As the second stage of the project, we plan to follow-up with another semi-structured interview or focus group workshop that will help capture a richer insight into your thoughts and feelings of a valued place in a changing world. If you are interested in taking part in this last stage of the project, kindly share your e-mail address or let the interviewer know at the end of the survey. The e-mail address you provide for this purpose will only be accessible to the research team and will only be used to invite you to participate in future parts of this study or for any other purpose you consent to at the end of the survey. Note that in these future stages, you will receive compensation for your time.

How can I find out more about this survey?

If you would like to find out more about the study, either before or after completion, please contact the research team leaders (see contact details below). If you are interested in the results of this study, please provide your e-mail at the end of the survey if you haven't already done so. Once this study is complete, we will provide you with a project summary and you will have the opportunity to provide opinions about the results. The e-mail address you provide for this purpose will only be accessible to the research team and will only be used to send you the study results or for any other purpose you consent to at the end of the survey.

Ethical clearance and contacts

This study has been approved by the University of Tasmania Human Research Ethics Committee (HREC). If you have concerns or complaints about the conduct of this study, you can contact the Executive Officer of the HREC on +61 3 6226 6254 or email human.ethics@utas.edu.au. The Executive Officer is the person nominated to receive complaints from research participants. You will need to quote H0029693.

How can I agree to be involved?

If you would like to proceed, please read the statement of consent below and click on the button at the bottom of this page. You must be 18 years or older to participate.

Thank you for considering participation in this research,

Edith Shum, Vanessa Adams, Georgina Gurney

Edith Shum | edith.shum@utas.edu.au
Vanessa Adams | vm.adams@utas.edu.au
Georgina Gurney | georgina.gurney@utas.edu.au

Unique ID: ___________

Consent

Please tear this page and keep it for your records

The Role of Species on your Connection to Place

Research Team:
Vanessa Adams, Supervisor, School of Geography, Planning & Spatial Sciences, University of Tasmania
Georgina Gurney, Associate Investigator, School of Geography, Planning & Spatial Sciences, University of Tasmania
Edith Shum, PhD Student, School of Geography, Planning & Spatial Sciences, University of Tasmania

P: +61 3 6226 1905
E: edith.shum@utas.edu.au

Participant Consent Statement

By continuing with this survey, I confirm that I have read and understood the information sheet, am 18 years or older, and in particular:

- I understand that I will not be compensated if fraudulent behaviour is detected such as completing the survey multiple times or indicating you qualify for participation (such as living in the region) fraudulently.
- I understand the information provided will stay anonymous and coded so that I will not be identified as a participant.
- I understand that the information I provide will only be accessed by members of the research team and used for the purposes outlined above. The data will be stored securely in University of Tasmania premises by the research team, analysed and then archived.
- I understand that the approximate place of residence information I provide will be confidential, that I or my residence will not be individually identifiable, and that this information will only be used for the following purposes: determining spatial representativeness of population sample; and ensuring data quality.
- I will not be personally identified in any publications resulting from the study.
- I understand that I may ask for part or all of the information provided by me to be removed from the study without penalty or explanation.
- I understand that the information I provide for this research will be treated confidentially and used for the following purposes: to produce scientific reports, papers, conference presentations and general media.
- I understand that my participation in the project is entirely voluntary and that I am free to withdraw from the study at any time and without having to provide a reason for my withdrawal.
- I have been provided with contact details of the investigating officers and understand that I can contact them at any point during the study. I have also been provided with the contact details of an independent research integrity advisory at UTAS should I wish to raise any concerns or complaints about the conduct of the research.
- I have agreed to participate in the above project being conducted by the University of Tasmania (UTAS).
- I have been provided with information about the project and had any questions regarding my participation and any associated risks and benefits answered to my satisfaction.
- I understand that my involvement in this research will include an online survey and interview that will be recorded and take approximately 20 to 30 minutes, with the potential for a follow-up interview should I express interest in participating in future parts of this study.

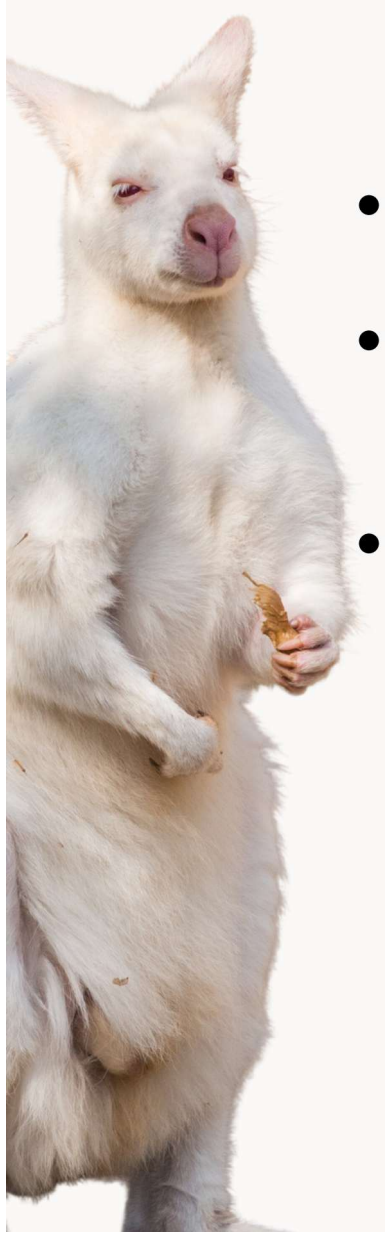

Once you've completed the survey, please place it in the prepaid envelope provided and drop it off at any post office.

To begin, let's first understand your general connection to Bruny Island.
To what extent do you agree with the following statements?

	Strongly Disagree	Disagree	Neutral	Agree	Strongly Agree
I feel like **Bruny Island** is a part of me	○	○	○	○	○
**Bruny Island** is very special to me	○	○	○	○	○
I identify strongly with **Bruny Island**	○	○	○	○	○
I am very attached to **Bruny Island**	○	○	○	○	○

	Strongly Disagree	Disagree	Neutral	Agree	Strongly Agree
Visiting **Bruny Island** says a lot about who I am	○	○	○	○	○
**Bruny Island** means a lot to me	○	○	○	○	○
**Bruny Island** is the best place for what I like to do	○	○	○	○	○
No other place can compare to **Bruny Island**	○	○	○	○	○

	Strongly Disagree	Disagree	Neutral	Agree	Strongly Agree
I get more satisfaction out of being in **Bruny Island** than any other place	○	○	○	○	○
Doing what I do in the **Bruny Island** is more important to me than doing it in other place	○	○	○	○	○
I would not substitute any other area for doing the types of things that I do at **Bruny Island**	○	○	○	○	○

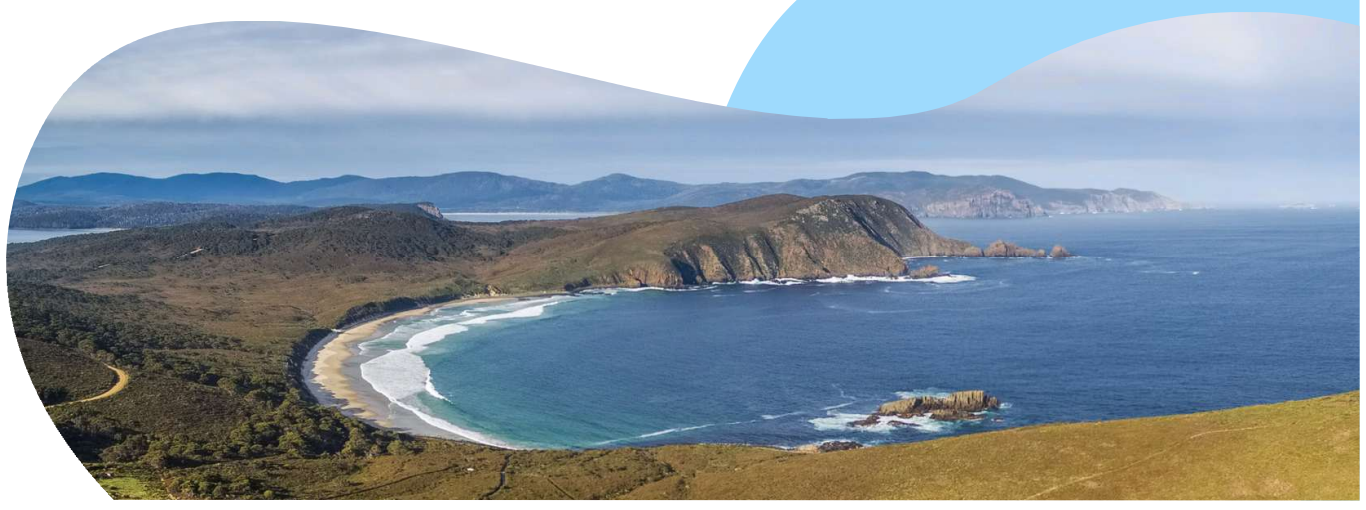

While we may feel a strong connection to Bruny Island as a whole, there can be specific places on Bruny that are more meaningful to us.

Take the Neck for example, an iconic landmark of Bruny. To explore your connection to the Neck, let's revisit the same series of questions, this time focusing on the Neck specifically.

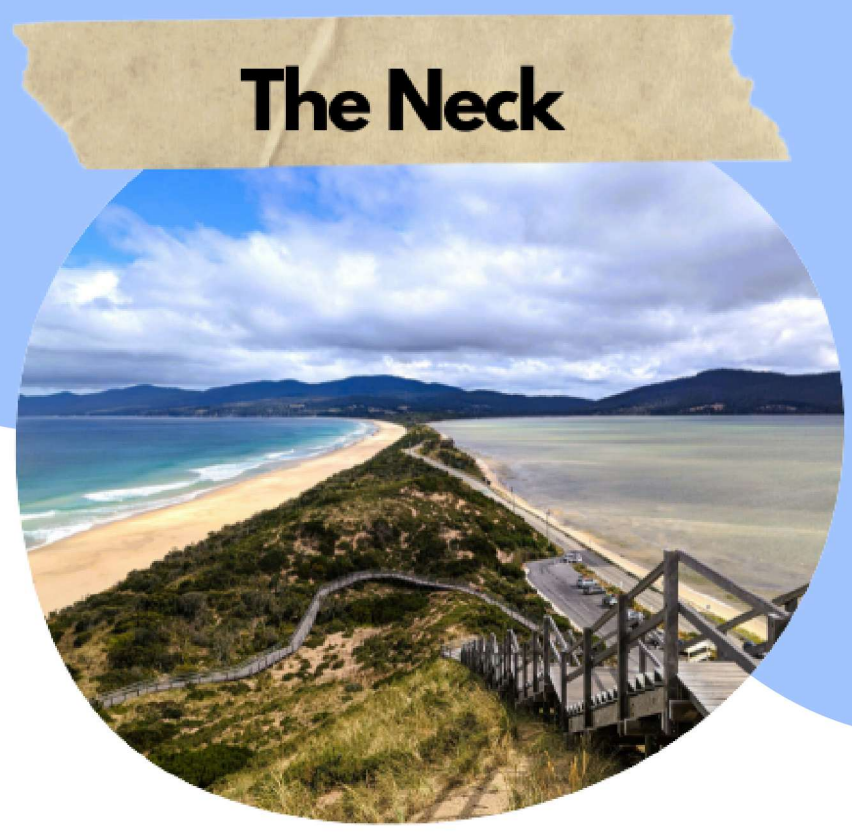

To what extent do you agree with the following statements?

	Strongly Disagree	Disagree	Neutral	Agree	Strongly Agree
I feel like **The Neck** is a part of me	○	○	○	○	○
**The Neck** is very special to me	○	○	○	○	○
I identify strongly with **The Neck**	○	○	○	○	○
I am very attached to **The Neck**	○	○	○	○	○

	Strongly Disagree	Disagree	Neutral	Agree	Strongly Agree
Visiting **The Neck** says a lot about who I am	○	○	○	○	○
**The Neck** means a lot to me	○	○	○	○	○
**The Neck** is the best place for what I like to do	○	○	○	○	○
No other place can compare to **The Neck**	○	○	○	○	○

	Strongly Disagree	Disagree	Neutral	Agree	Strongly Agree
I get more satisfaction out of being in **The Neck** than any other place	○	○	○	○	○
Doing what I do in the **The Neck** is more important to me than doing it in other place	○	○	○	○	○
I would not substitute any other area for doing the types of things that I do at **The Neck**	○	○	○	○	○

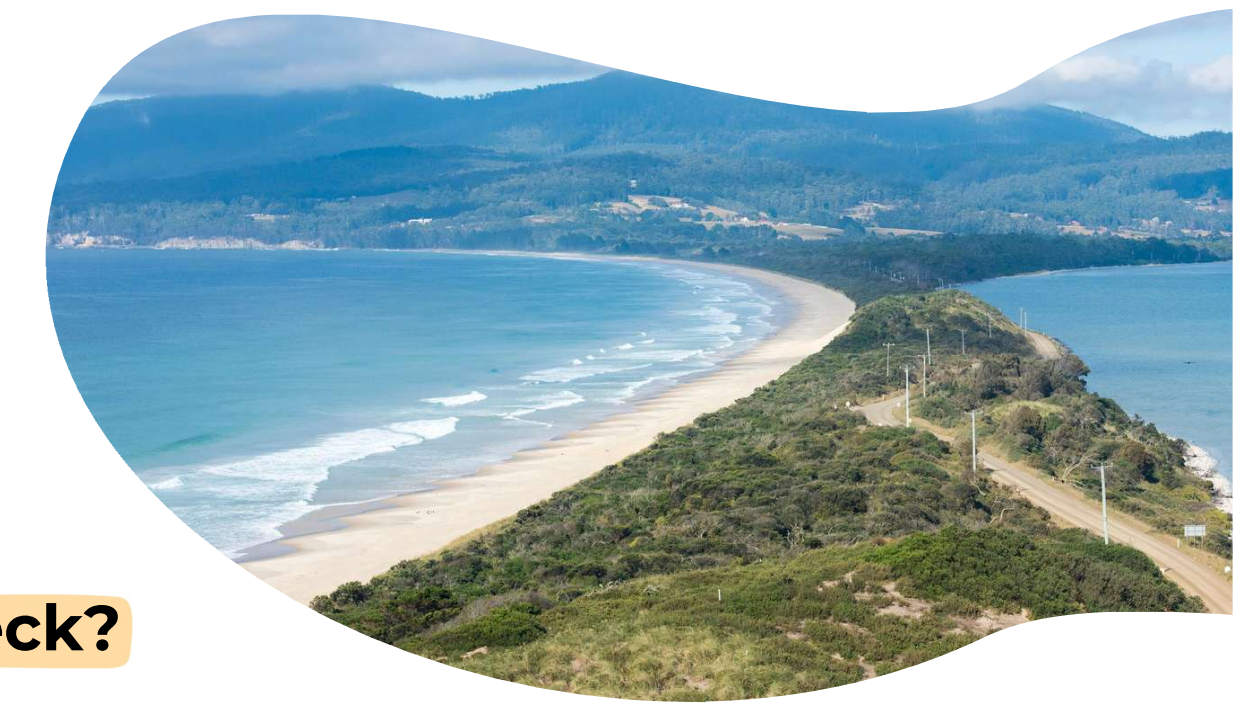

What about your personal connection to The Neck? Please consider the following statements:

	Strongly Disagree	Disagree	Neutral	Agree	Strongly Agree
**The Neck** reminds me of a significant event in my life	○	○	○	○	○
**The Neck** reflects the type of person I am	○	○	○	○	○
**The Neck** is such a spiritual place	○	○	○	○	○
**The Neck** makes me feel connected to this community	○	○	○	○	○

With the Neck in mind, take a moment to consider the fairy penguins making their way up to the beach or the flocks of shearwaters gliding in from the sea. The following set of questions will ask you to reflect on your connection with fairy penguins commonly observed at the Neck.

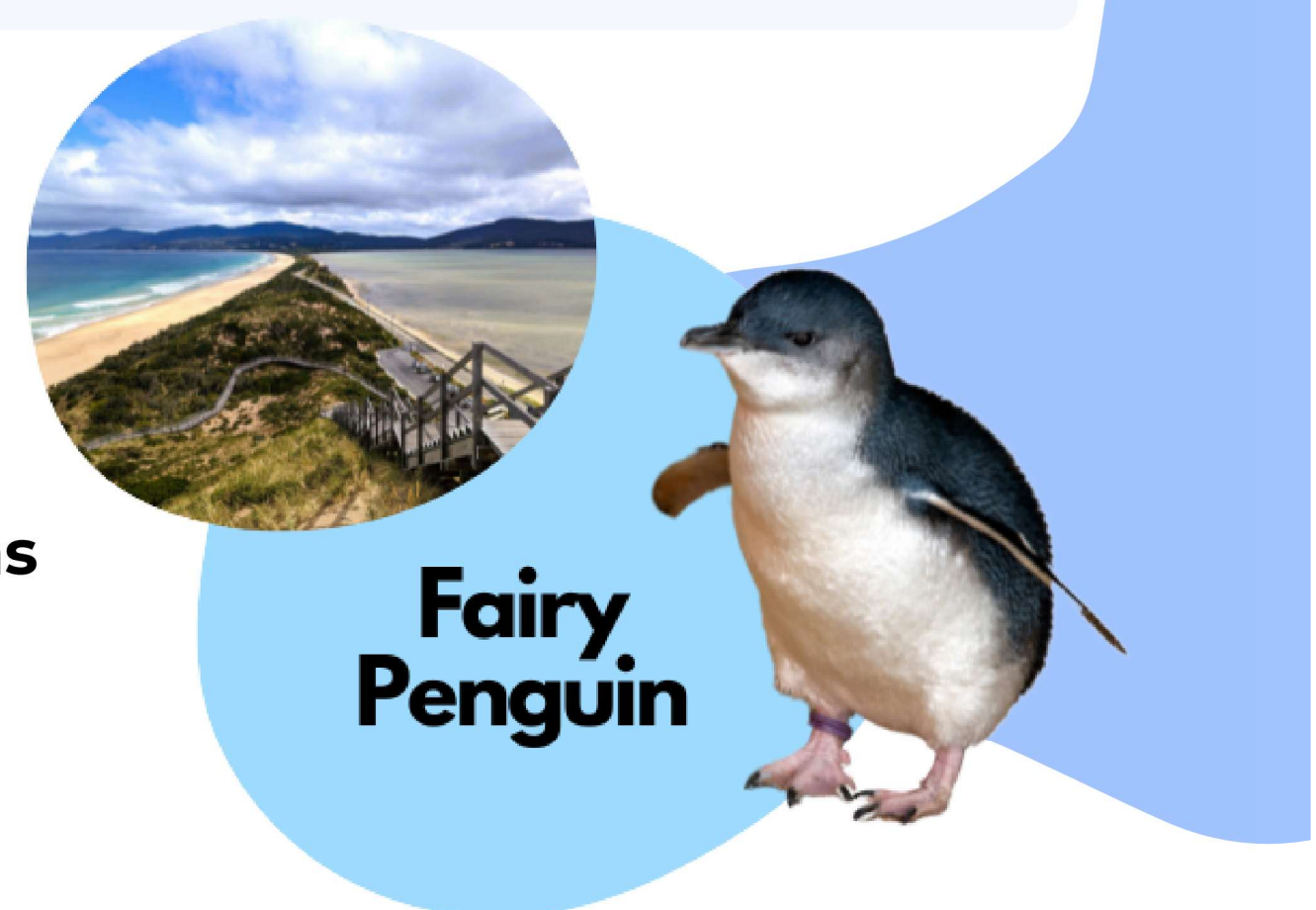

First let's consider the role fairy penguins at the Neck for the following statements:

	Strongly Disagree	Disagree	Neutral	Agree	Strongly Agree
**Fairy penguins** are like 'kin' or family to me, so how we treat them here at **the Neck** matters	○	○	○	○	○
How I care for **fairy penguins** reflects my sense of responsibility to and stewardship for **the Neck**	○	○	○	○	○
Other people often disagree about my concerns with **fairy penguins** at **the Neck**	○	○	○	○	○

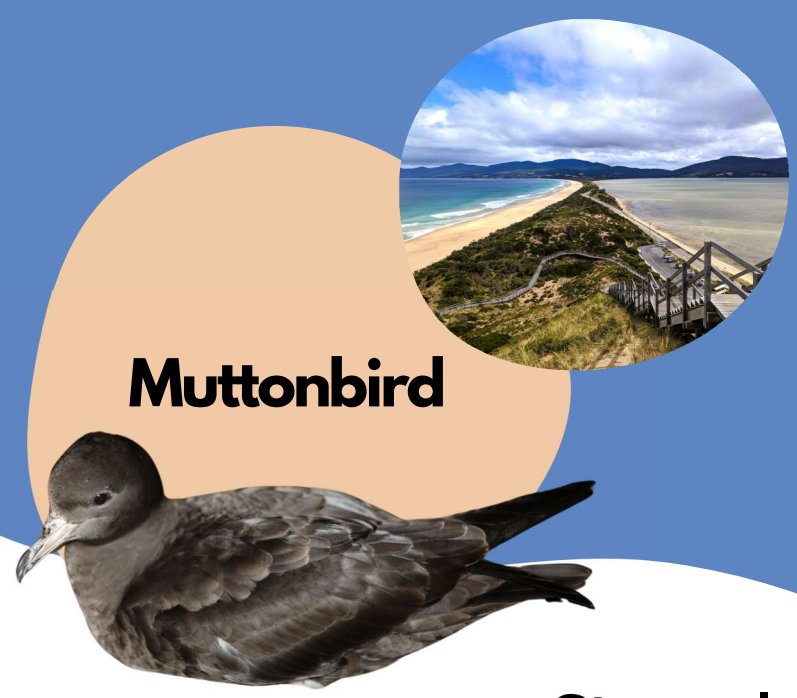

Now let's consider the role of muttonbirds at the Neck for the following statements:

	Strongly Disagree	Disagree	Neutral	Agree	Strongly Agree
**Muttonbirds** are like 'kin' or family to me, so how we treat them here at **the Neck** matters					
How I care for **muttonbirds** reflects my sense of responsibility to and stewardship for **the Neck**					
Other people often disagree about my concerns with **muttonbirds** at **the Neck**					

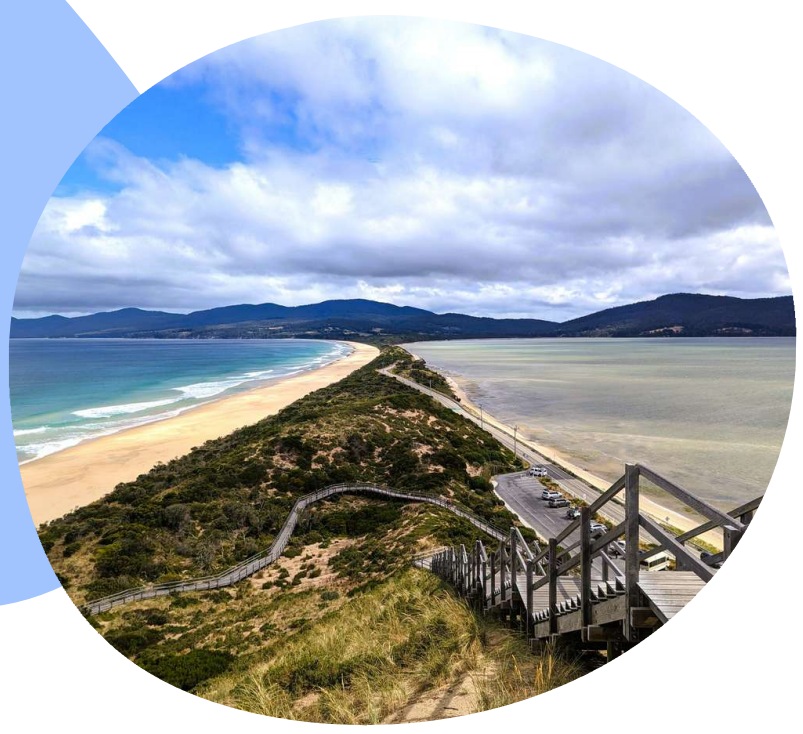

Now let's explore the processes involved in these relationships a bit more. Please only consider the Neck again for the following statements:

	Not at all	Slightly	Neutral	Often	Always
I feel a sense of emotional attachment to **the Neck**					
I feel strong, positive feelings for **the Neck**					
**The Neck** makes me recall my memories					
**The Neck** reminds me of the environment of my childhood					
**The Neck** is a place to go on special occasions					
If I am away from the Neck for an extended period of time, I will make an effort to return to or visit **the Neck**					

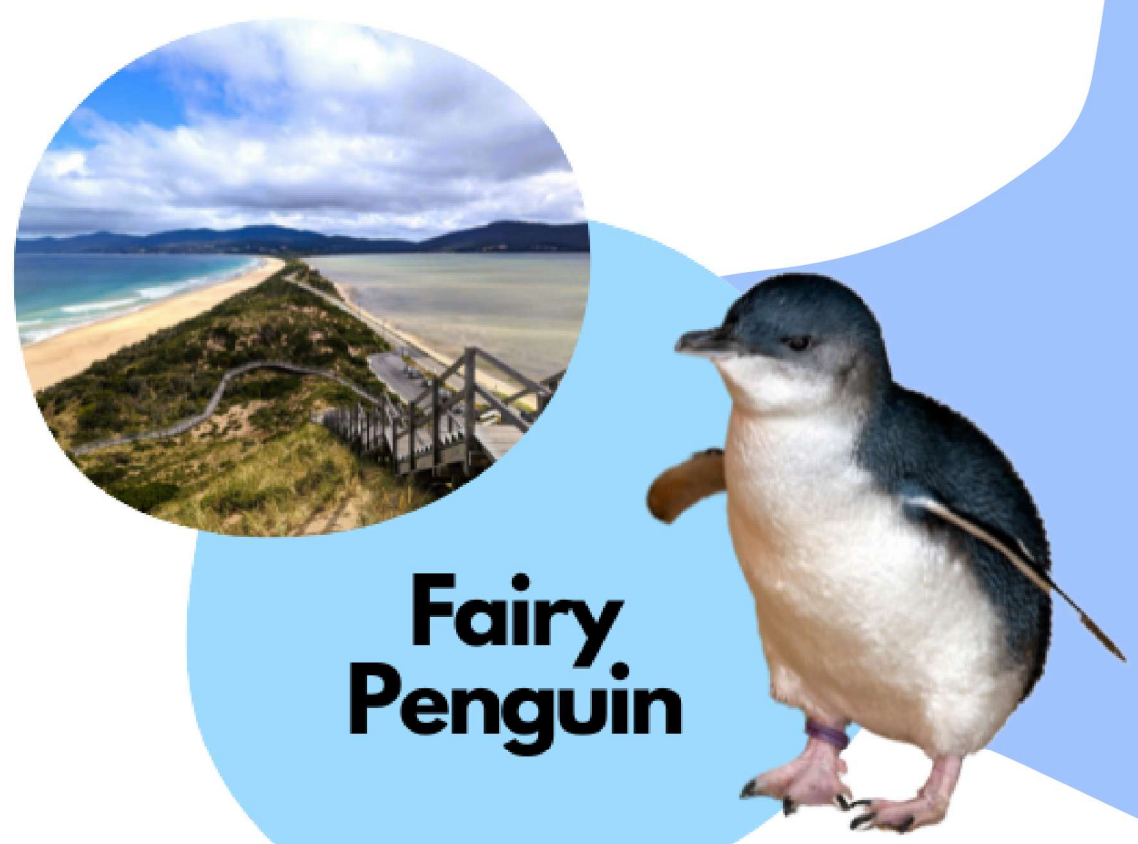

Please answer the following questions in regards to fairy penguins at the Neck

	Not at all	Slightly	Neutral	Often	Always
The presence of **fairy penguins** within **the Neck** plays a role in enhancing both biological diversity and ecosystem services	○	○	○	○	○
The presence of **fairy penguins** at **the Neck** have a positive economic impact	○	○	○	○	○
I am reminded about the **fairy penguins**' cultural significance at **the Neck**	○	○	○	○	○
**Fairy penguins** make **the Neck** beautiful	○	○	○	○	○
There is sacred, religious or spiritual significance of **fairy penguins** at **the Neck**	○	○	○	○	○

	Not at all	Slightly	Neutral	Often	Always
I am reminded about the **fairy penguins** history at **the Neck**	○	○	○	○	○
I remember fond stories or anecdotes of **fairy penguins** at **the Neck** told from relatives, family, or friends.	○	○	○	○	○
When I see **fairy penguins**, I often think of other species that could also be at **the Neck**	○	○	○	○	○
I always hope to see **fairy penguins** around when I am out at **the Neck**	○	○	○	○	○

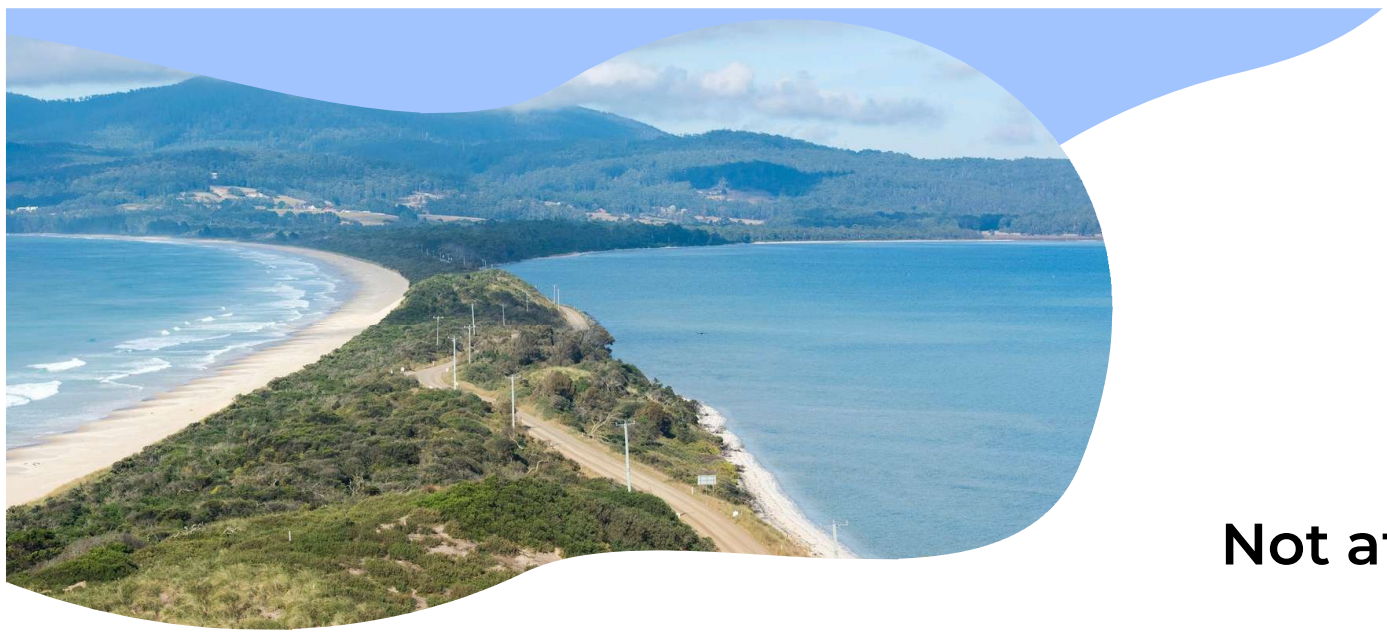

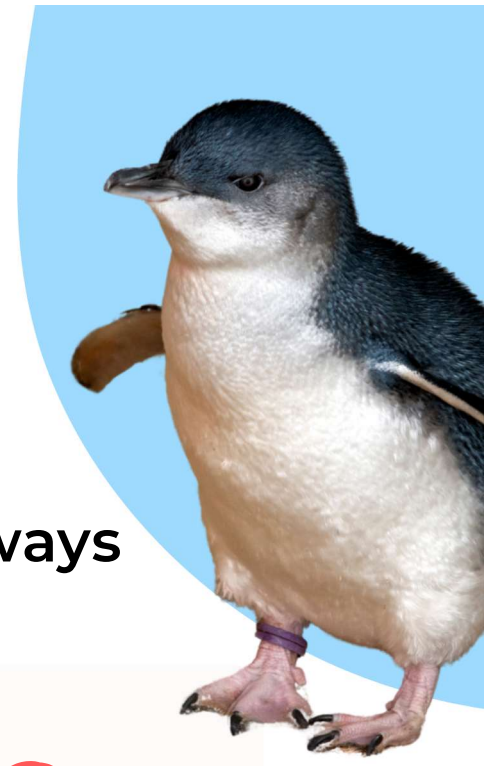

	Not at all	Slightly	Neutral	Often	Always
I feel more connected to **the Neck** when I hear **fairy penguins** in this environment	○	○	○	○	○
I am overcome with emotions when I interact with **fairy penguins** at **the Neck**	○	○	○	○	○
I would feel less attached to **the Neck** if **fairy penguins** that lived here disappeared	○	○	○	○	○
I am well-informed about **fairy penguins** present at **the Neck**	○	○	○	○	○
The role of **fairy penguins** in sustaining **the Neck** is important	○	○	○	○	○

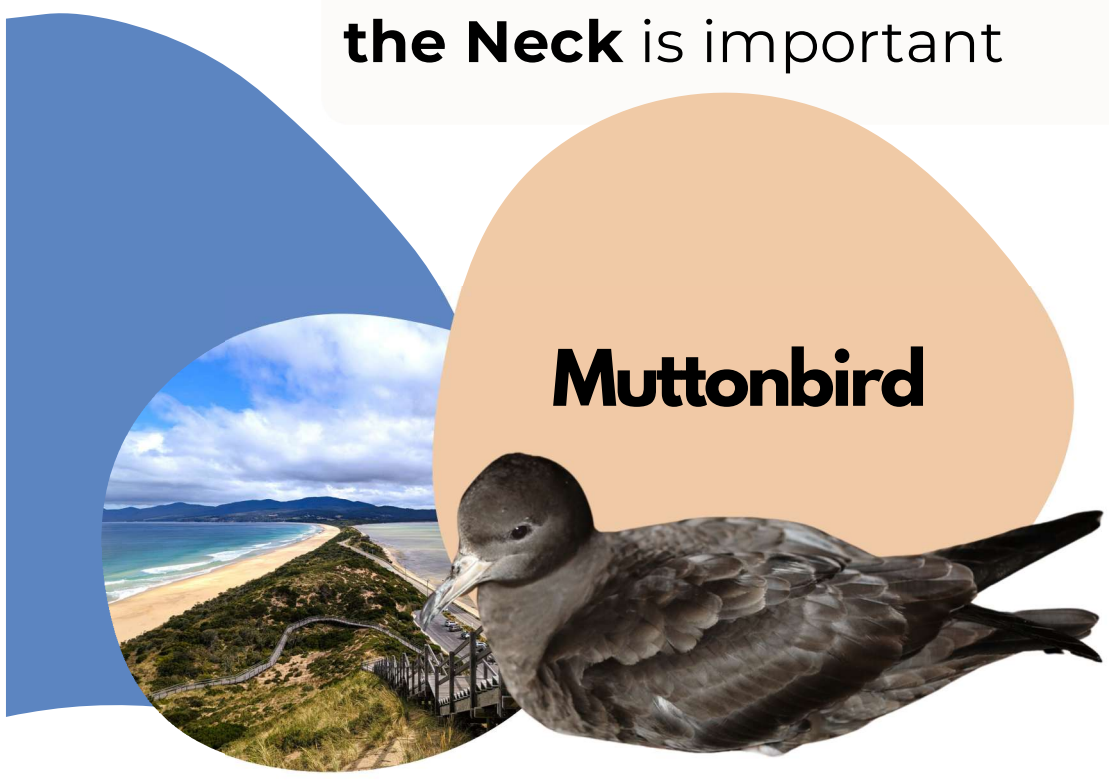

Let’s focus on muttonbirds at the Neck for the following statements:

	Not at all	Slightly	Neutral	Often	Always
The presence of **muttonbirds** within **the Neck** plays a role in enhancing both biological diversity and ecosystem services	○	○	○	○	○
The presence of **muttonbirds** at **the Neck** have a positive economic impact	○	○	○	○	○
I am reminded about the **muttonbirds’** cultural significance at **the Neck**	○	○	○	○	○

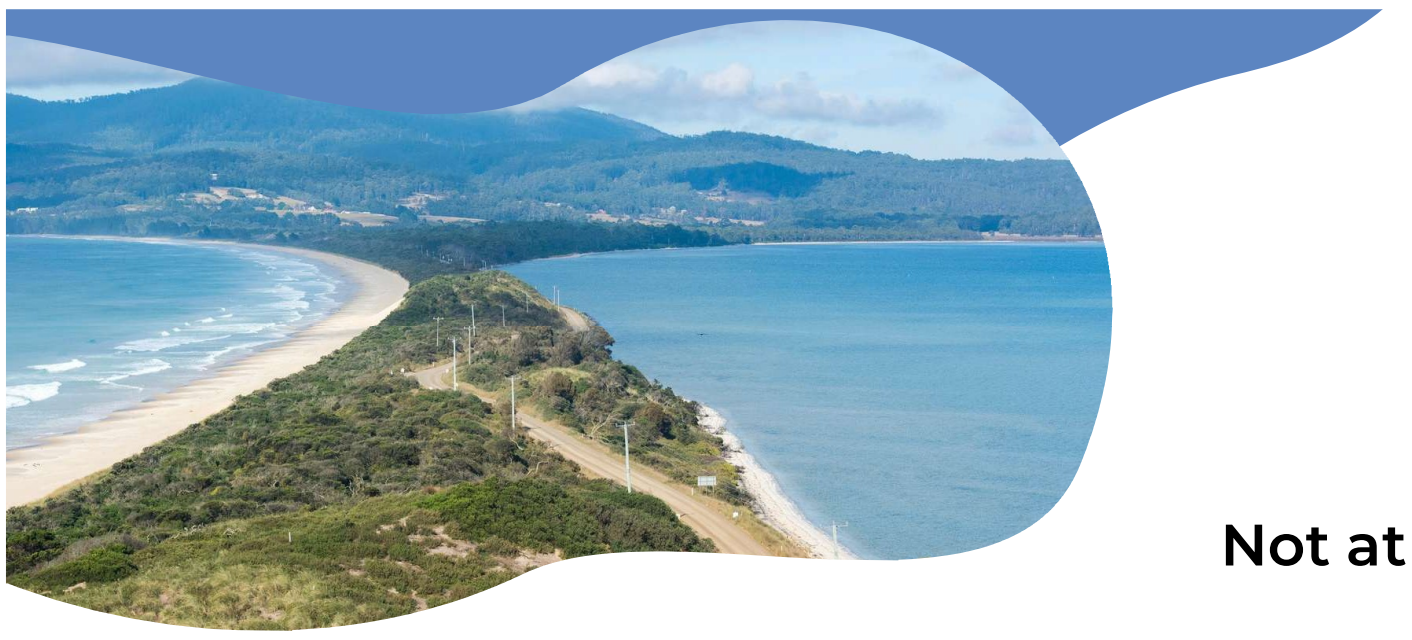

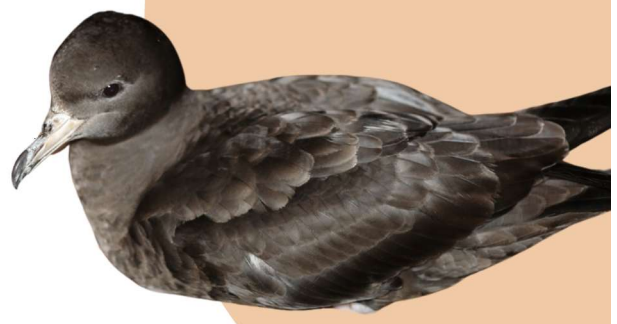

	Not at all	Slightly	Neutral	Often	Always
**Muttonbirds** make **the Neck** beautiful	○	○	○	○	○
There is sacred, religious or spiritual significance of **muttonbirds** at **the Neck**	○	○	○	○	○
I am reminded about the **muttonbirds** history at **the Neck**	○	○	○	○	○
I remember fond stories or anecdotes of **muttonbirds** at **the Neck** told from relatives, family, or friends.	○	○	○	○	○
When I see **muttonbirds**, I often think of other species that could also be at **the Neck**	○	○	○	○	○
I always hope to see **muttonbirds** around when I am out at **the Neck**	○	○	○	○	○
I feel more connected to **the Neck** when I hear **muttonbirds** in this environment	○	○	○	○	○
	**Not at all**	**Slightly**	**Neutral**	**Often**	**Always**
I am overcome with emotions when I interact with **muttonbirds** at **the Neck**	○	○	○	○	○
I would feel less attached to **the Neck** if **muttonbirds** that lived here disappeared	○	○	○	○	○
I am well-informed about **muttonbirds** present at **the Neck**	○	○	○	○	○
The role of **muttonbirds** in sustaining **the Neck** is important	○	○	○	○	○

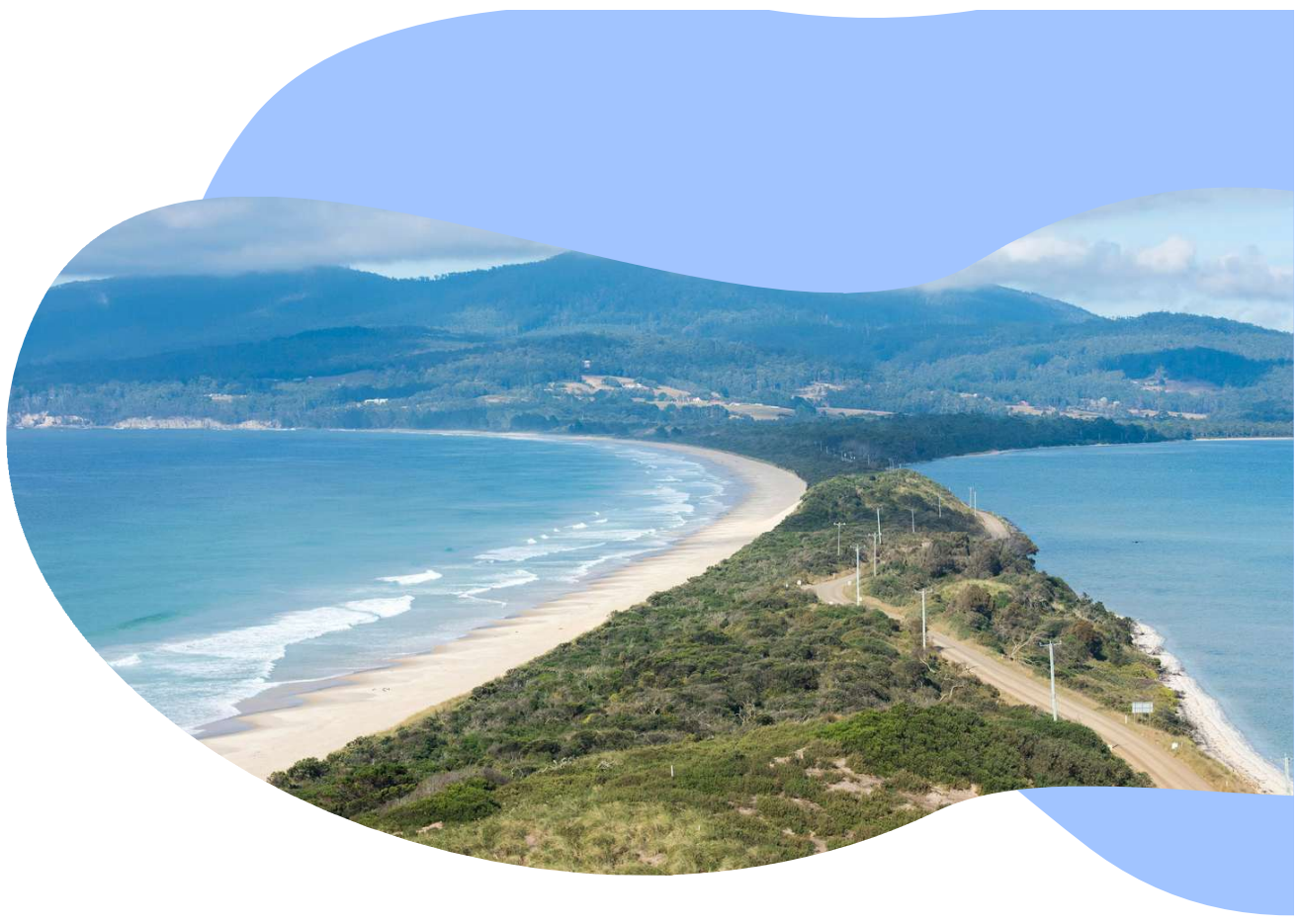

Finally, let's explore the features of the place itself that make the Neck so special to us.

Please only consider the Neck again for the following statements:

	Strongly Disagree	Disagree	Neutral	Agree	Strongly Agree
I feel a sense of togetherness with others who also like **the Neck**	○	○	○	○	○
Many of my friends/family prefer **the Neck** compared to other places	○	○	○	○	○
**The Neck** has become an important landmark on Bruny Island	○	○	○	○	○
**The Neck** is of high environmental quality	○	○	○	○	○
I find **the Neck** beautiful	○	○	○	○	○

How often do you visit the Neck?

○ Daily ○ Monthly ○ Annually

○ Weekly ○ Semi-annually ○ Less than once a year

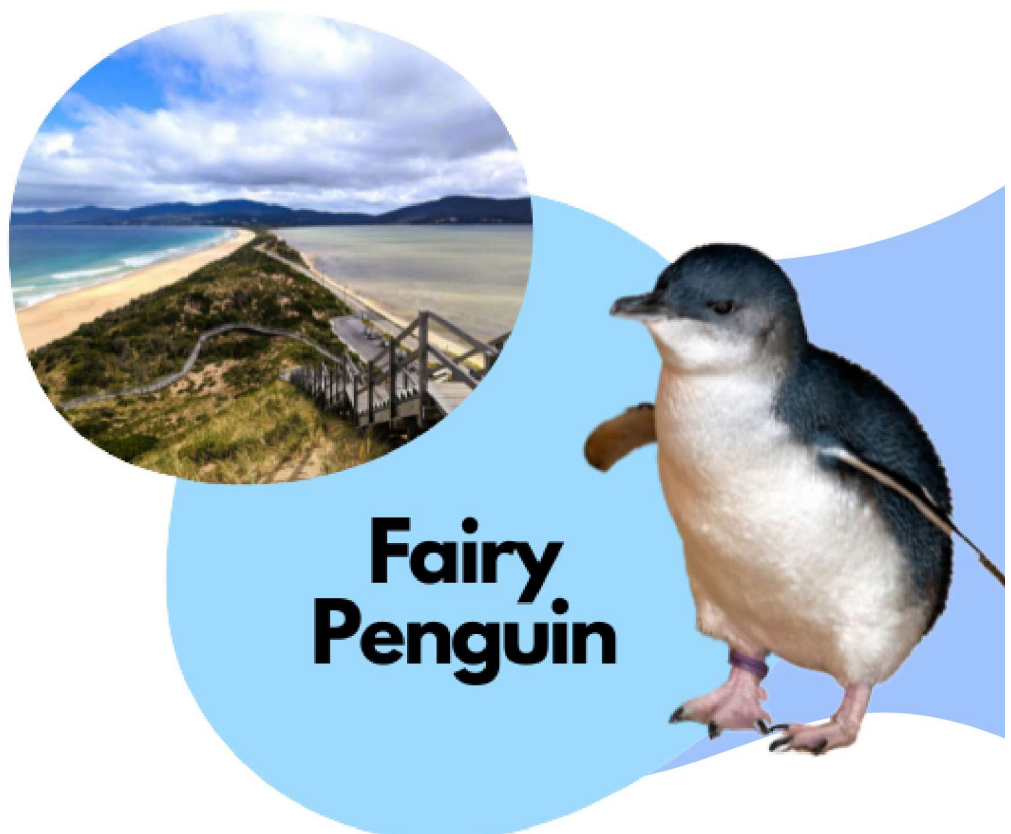

Finally, to what extent do you agree with the following statements:

	Strongly Disagree	Disagree	Neutral	Agree	Strongly Agree
**Fairy penguins** are a symbolic or iconic feature of **the Neck**	○	○	○	○	○
	**Not at all**	**Slightly**	**Neutral**	**Important**	**Extremely Important**
The native status of **fairy penguins** at **the Neck** matter to me.	○	○	○	○	○

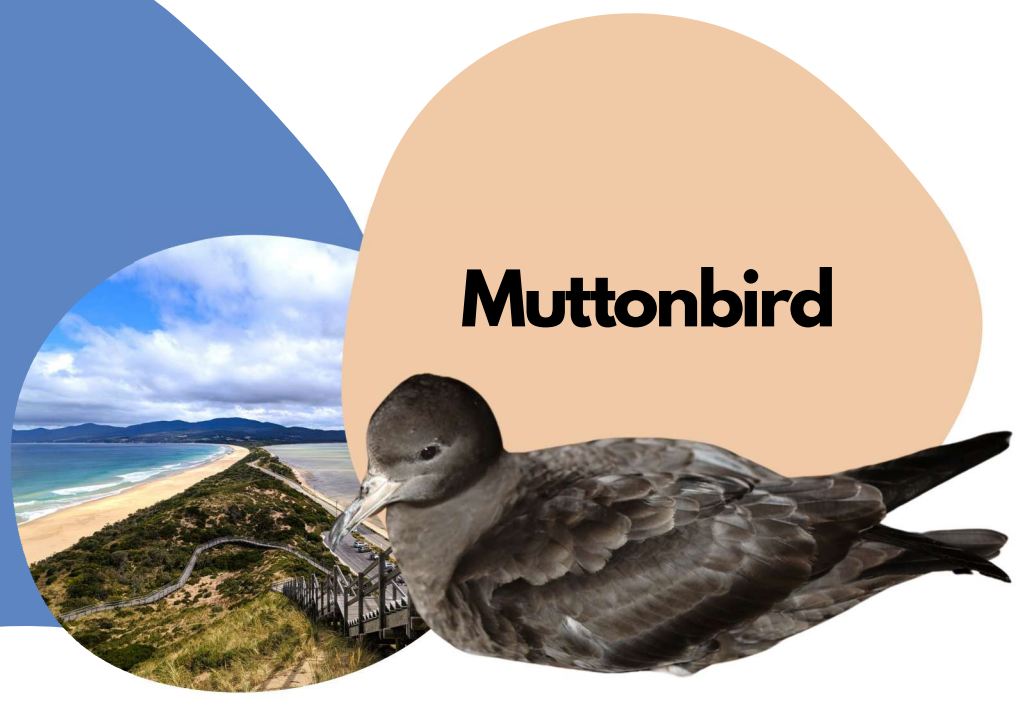

To what extent do you agree with the following statements?

	Strongly Disagree	Disagree	Neutral	Agree	Strongly Agree
**Muttonbirds** are a symbolic or iconic feature of **the Neck**	○	○	○	○	○
	**Not at all**	**Slightly**	**Neutral**	**Important**	**Extremely Important**
The native status of **muttonbirds** at **the Neck** matter to me.	○	○	○	○	○

To wrap up, please consider the following 5 questions designed to understand your relationship between species, people, and special places.

Each question will present various sets of circles, and you'll be asked to select the set that best represents your relationship to the label within the circles. The closer the circles are to each other, the more connected you feel; the farther apart the circles, the less connected you are.

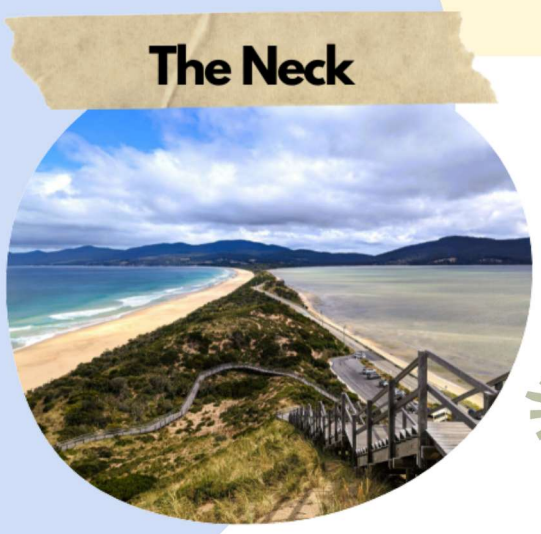

1. Please draw a rectangle around the set of circles that best describes your relationship to **the Neck**

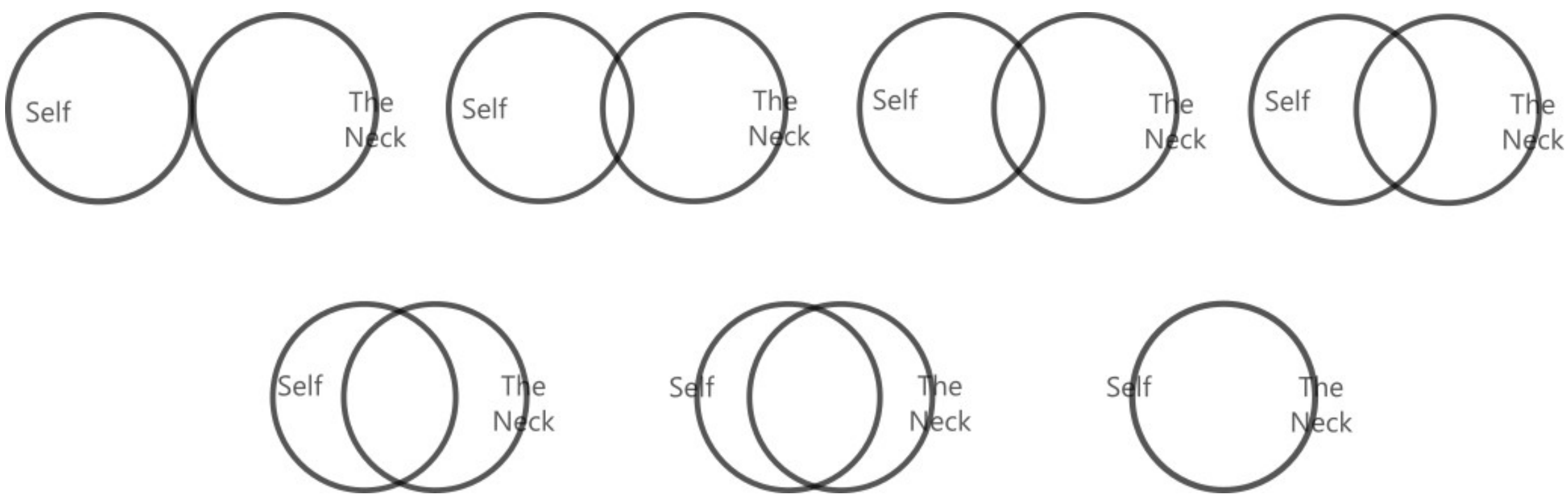

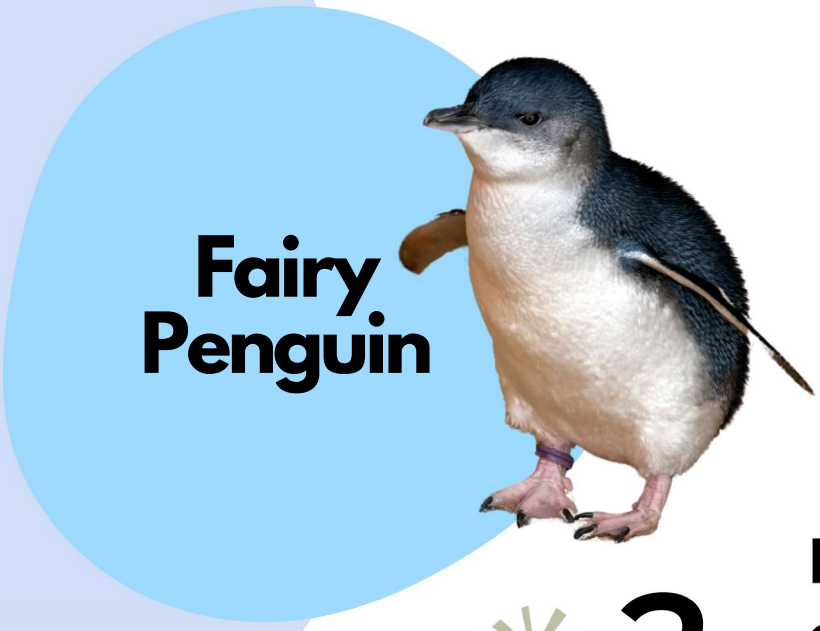

2. Please draw a rectangle around the set of circles that best describes your relationship to fairy penguins

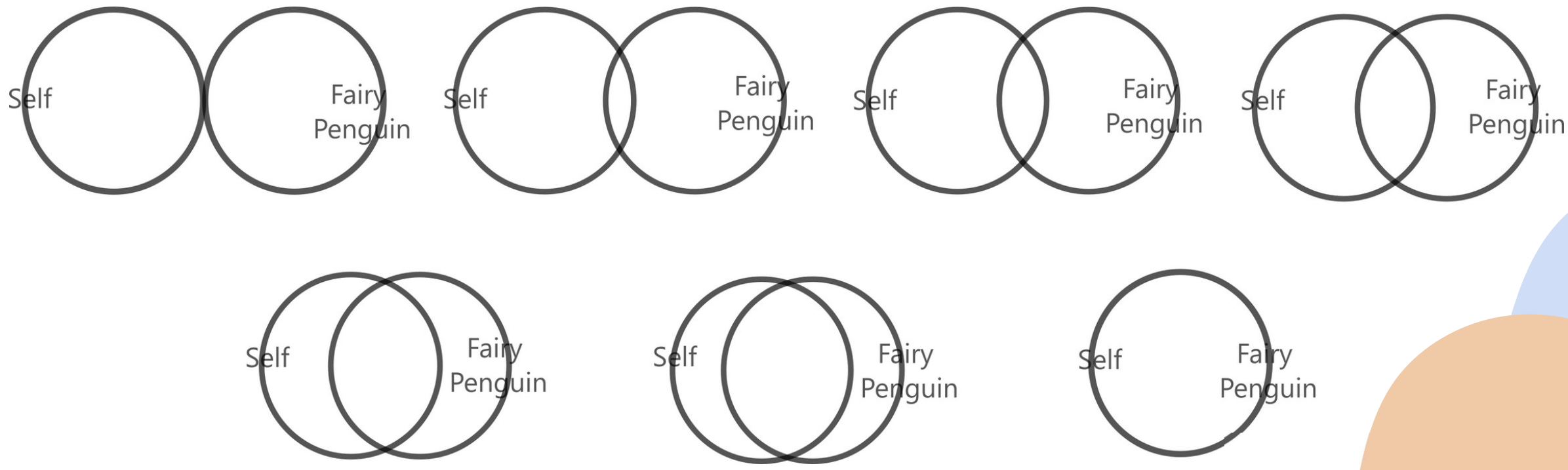

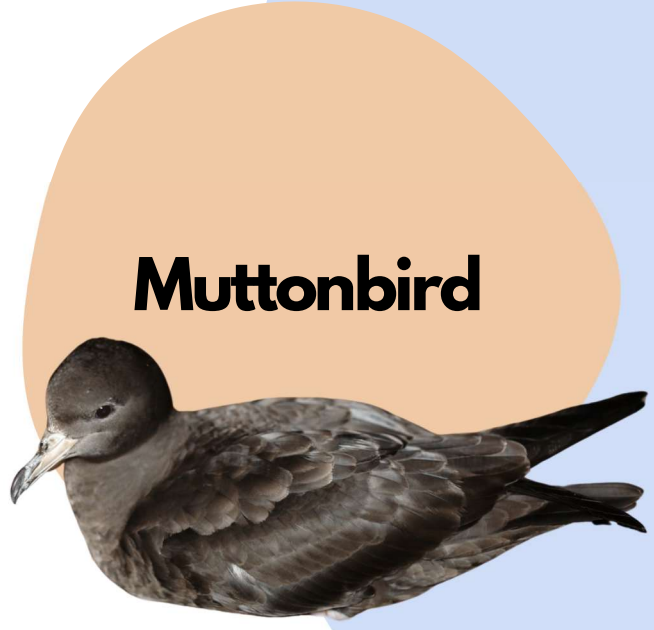

3. Please draw a rectangle around the set of circles that best describes your relationship to muttonbirds

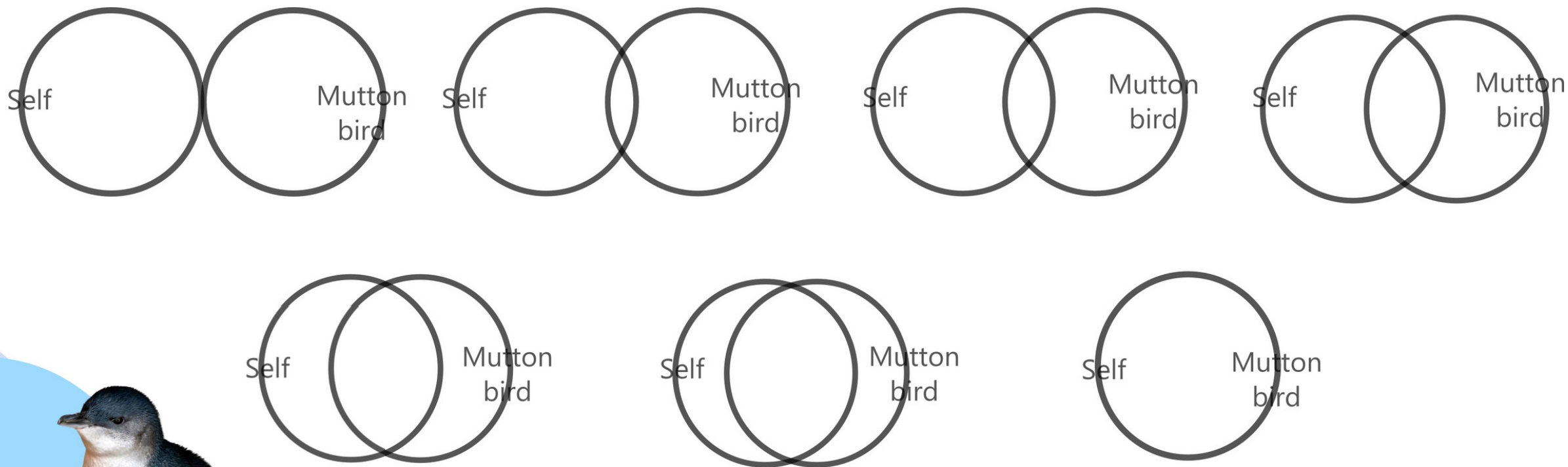

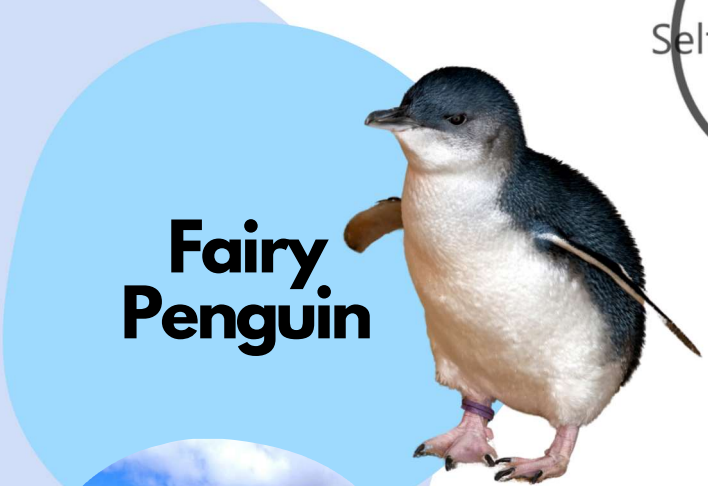

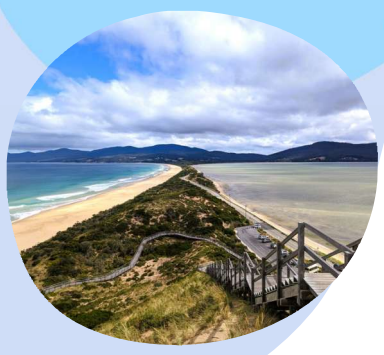

4. Please draw a rectangle around the set of circles that best describes your association between on fairy penguins and the Neck.

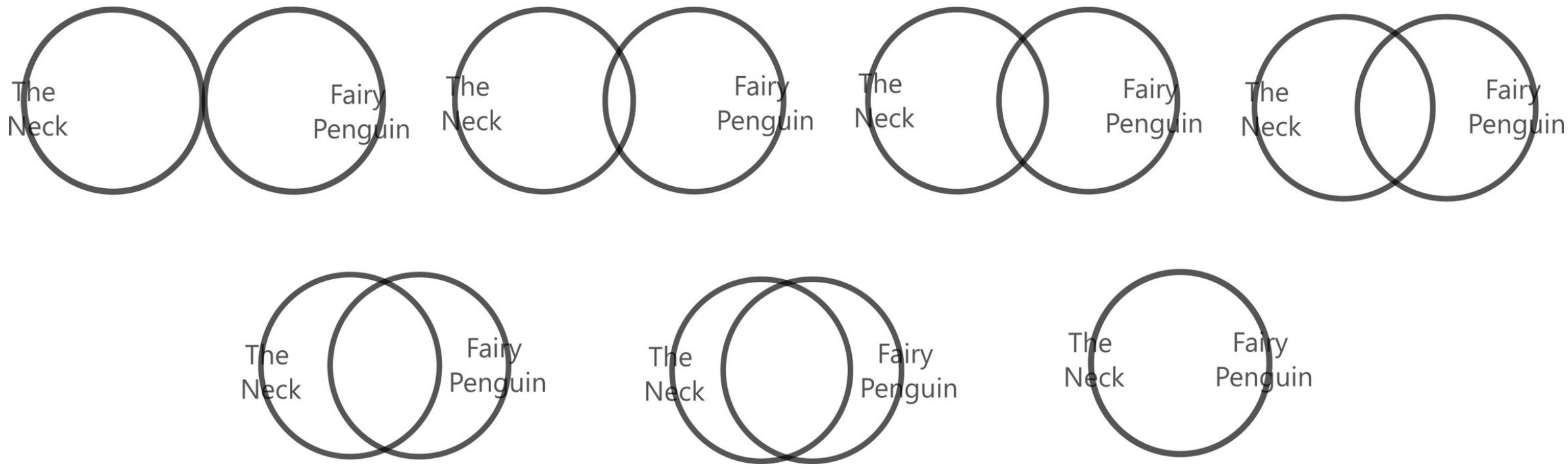

Muttonbird

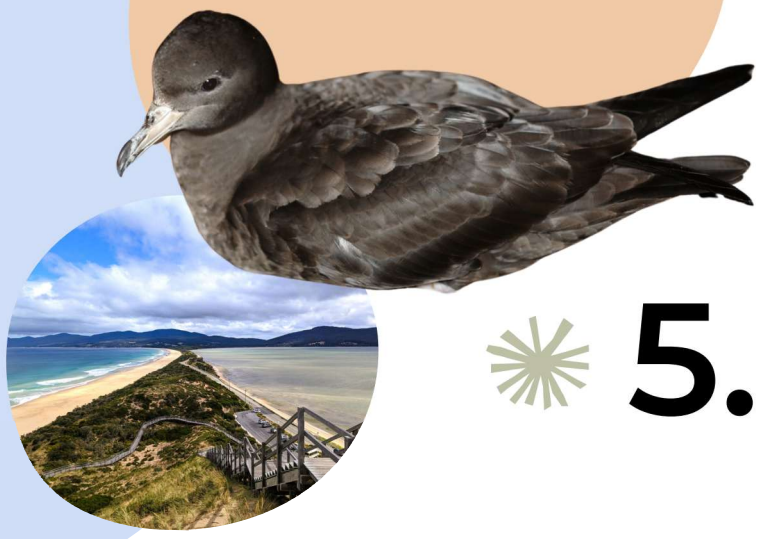

5. Please draw a rectangle around the set of circles that best describes your association between muttonbirds and the Neck

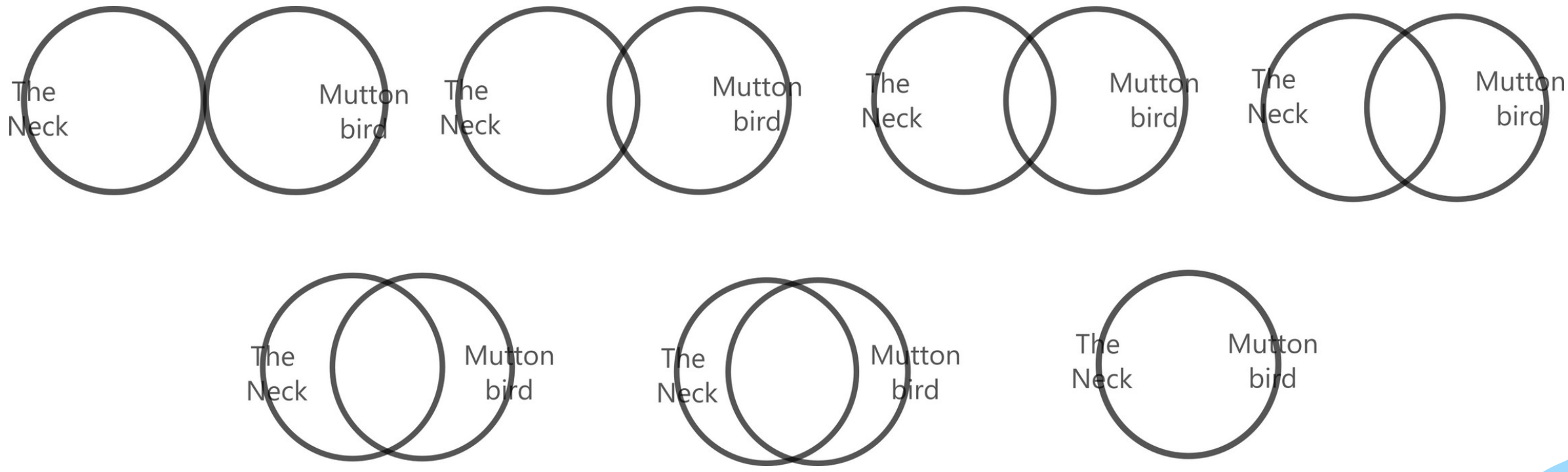

Lastly, please answer the following demographic questions

Which of the following applies to you (please select all that apply)?

- ○ My permanent residence is located on Bruny Island
- ○ I have a holiday home (i.e. shack) on Bruny Island
- ○ I have close family on Bruny Island
- ○ My main livelihood is based on Bruny Island (e.g. a business, farm, fishery, work)
- ○ Other: ____

Please identify the suburb/township closest to your primary connection on Bruny Island:

What is the postcode of your primary residence?

How many years have you lived, worked, owned property, or held a connection to Bruny Island?

- Less than one year
- 1 to 3 years
- >3 to 5 years
- >5 to 10 years
- More than 10 years
- All my life

What is your age? If you <*prefer not to say*>, skip to the next question

What is the highest degree of level of education you have completed?

- Primary school
- Highschool
- Undergraduate degree
- Postgraduate degree
- TAFE/Vocational qualification or Trade School
- Other: ___________________________________

How do you identify?

- Male
- Female
- Non-binary/third gender
- Prefer to self-describe: ___________________________________
- Prefer not to say

What is your main occupation at the moment?

- Self-employed/entrepreneur
- Employee
- Apprentice
- Student
- Unemployed
- Full time caretaking for kids or other family members
- Retired
- Other: ___________________________________

To which of the following industries do you belong?

- Accommodation and food services
- Administrative and support services
- Agriculture, forestry, fishing
- Arts and recreation services
- Construction
- Education and training
- Electricity, gas, water, and waste services
- Finance and insurance services
- Finance and insurance services
- Healthcare and social assistance
- Information, media, and telecommunications
- Manufacturing
- Mining
- Professional, scientific, and technical services
- Public administration and safety
- Public Official/Government
- Retail trader
- Rental, hiring, and real estate
- Transport, postal, and warehousing
- Wholesale trader
- Other: ______________________________

Which of these describes your personal income last year?

- ◯ $0
- ◯ $1 to $ 9 999
- ◯ $10 000 to $24 999
- ◯ $25 000 to $49 999
- ◯ $50 000 to $74 999
- ◯ $75 000 to $99 999
- ◯ $100 000 to $149 999
- ◯ $150 000 and greater
- ◯ Prefer not to answer

Are you of Aboriginal or Torres Strait Islander Origin?

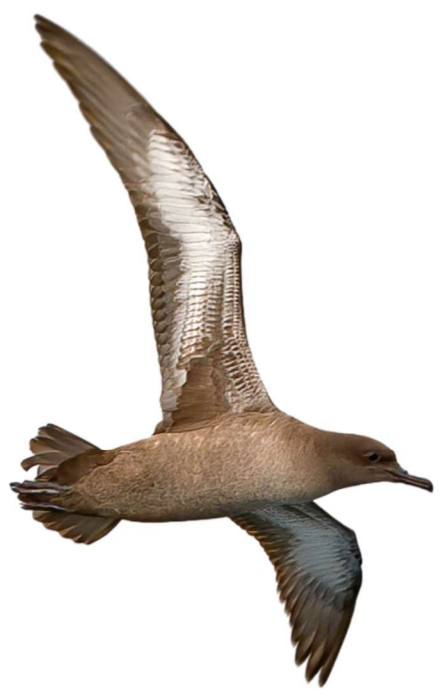

- ◯ Yes
- ◯ No

If yes, which group or community do you identify with?

If you're interested in the results, please provide your email address below:

Thank you for your time! 😊 As a token of appreciation, please provide your email address below for a chance to win a $100 digital gift voucher for delightful treats and drinks from the renowned Bruny Island Cheese Co.? 🧀🍻

This survey is the second phase of a wider project that seeks to explore how valued species influence our connection to places on Bruny Island. If you're interested in being involved in the final phase of this study, please express your interest below. You will be compensated for your time and can withdraw at any time of the project.

First Name:

Last Name:

Email Address:

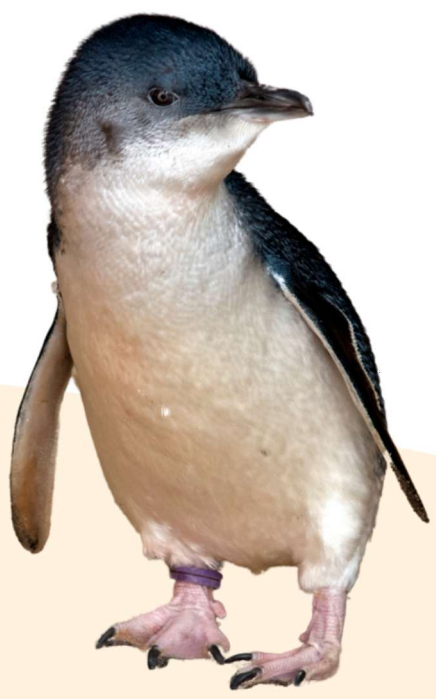

UNIVERSITY of TASMANIA

ESA

TASMANIAN LAND CONSERVANCY

connection to species on bruny island

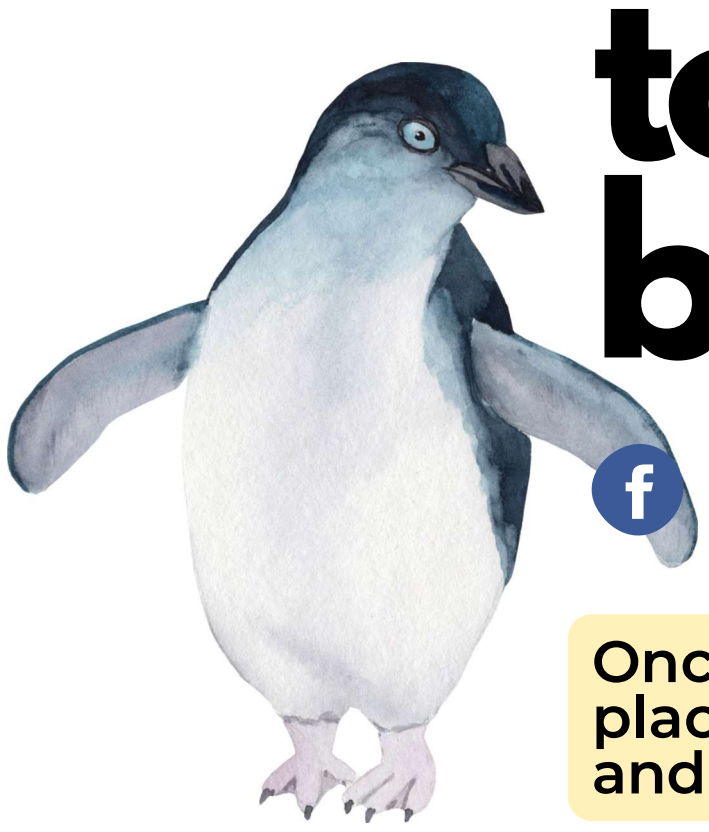

FOLLOW TO LEARN MORE

Once you've completed the survey, please place it in the prepaid envelope provided and drop it off at any post office.
