# Supplementary material for: Characterising people–species–place relationships
Source: Ambio. 2026 May 23;55(12):2905–20. doi: 10.1007/s13280-026-02411-0 (PMC13731557; doi:10.1007/s13280-026-02411-0)
Supplement: Supplementary file 2 [file 13280_2026_2411_MOESM2_ESM.pdf]

Supplementary Information: This Supplementary Information has not been peer reviewed.

Title: Characterising people-species-place relationships

Appendix S2: Interview questions grounded in the six key roles of species in place attachment were used to prompt participants to reflect on how fairy penguins and muttonbirds contribute to their connection to The Neck.

1. What do fairy penguins/muttonbirds mean to you? Do they carry any special meaning? What do they represent? Or, what significance do they hold for you?
    a. What words would you use to describe the value of fairy penguins/muttonbirds at The Neck for you? (e.g. iconic, symbolic, important)
2. What role do fairy penguins/muttonbirds play in your connection to The Neck?
    a. Please describe any childhood memories, or general memories or experiences, feelings, interactions, including sound, or smell about fairy penguins/muttonbirds at The Neck
    b. How did you acquire your knowledge about fairy penguins/muttonbirds at the Neck? (from reading, friends, experience)
3. Sometimes people visit places for various reasons. One is because of the way we interact with the landscape, such as going to The Neck to take a relaxing walk along the white sandy beaches. But it may also be because of your specific desires. For instance, I may go to The Neck because I want to see or experience something in particular. When you go to The Neck, what is your primary reason? Is it primarily driven by the presence of birds, or do you feel a deep connection with The Neck itself, and the birds are only an add-on to your overall experience?
    a. How much do fairy penguins/muttonbirds make up what The Neck means for you. Would the meaning of the Neck change for you without the presence of fairy penguins/muttonbirds. Would it still be the Neck to you?
4. Is there anything else you'd like to share?
